# Regional-Scale Declines in Productivity of Pink and Chum Salmon Stocks in Western North America

**DOI:** 10.1371/journal.pone.0146009

**Published:** 2016-01-13

**Authors:** Michael J. Malick, Sean P. Cox

**Affiliations:** School of Resource and Environmental Management, Simon Fraser University, Burnaby, British Columbia, Canada; Swedish University of Agricultural Sciences, SWEDEN

## Abstract

Sockeye salmon (*Oncorhynchus nerka*) stocks throughout the southern part of their North American range have experienced declines in productivity over the past two decades. In this study, we tested the hypothesis that pink (*O*. *gorbuscha*) and chum (*O*. *keta*) salmon stocks have also experienced recent declines in productivity by investigating temporal and spatial trends in productivity of 99 wild North American pink and chum salmon stocks. We used a combination of population dynamics and time series models to quantify individual stock trends as well as common temporal trends in pink and chum salmon productivity across local, regional, and continental spatial scales. Our results indicated widespread declines in productivity of wild chum salmon stocks throughout Washington (WA) and British Columbia (BC) with 81% of stocks showing recent declines in productivity, although the exact form of the trends varied among regions. For pink salmon, the majority of stocks in WA and BC (65%) did not have strong temporal trends in productivity; however, all stocks that did have trends in productivity showed declining productivity since at least brood year 1996. We found weaker evidence of widespread declines in productivity for Alaska pink and chum salmon, with some regions and stocks showing declines in productivity (e.g., Kodiak chum salmon stocks) and others showing increases (e.g., Alaska Peninsula pink salmon stocks). We also found strong positive covariation between stock productivity series at the regional spatial scale for both pink and chum salmon, along with evidence that this regional-scale positive covariation has become stronger since the early 1990s in WA and BC. In general, our results suggest that common processes operating at the regional or multi-regional spatial scales drive productivity of pink and chum salmon stocks in western North America and that the effects of these process on productivity may change over time.

## Introduction

The influence of environmental change on fish population productivity is a central problem in fisheries science [1]. Long time series of marine fish stock data indicate that population dynamics of exploited fish species are non-linear with high potential for abrupt and unpredictable shifts in response to environmental variability [2]. Large-scale shifts in productivity have been observed for a wide range of demersal and pelagic fish stocks, including North Atlantic cod (*Gadus morhua*)[3,4], Pacific salmon (*Oncorhynchus* spp.)[5,6], Pacific herring (*Clupea pallasi*)[7,8], and sardines [9,10]. Some productivity changes occur via abrupt and persistent regime shifts such as the three-fold increase in productivity observed for Bristol Bay sockeye salmon (*O*. *nerka*) after the 1976/77 climatic regime shift [5,11]. In other cases, such as several Atlantic cod stocks, productivity changes gradually over decades [4]. Regardless of the functional form of the productivity trends, observed changes in productivity often persist [12], potentially increasing management or conservation risks if the shifts are not anticipated or detected quickly because fisheries may need to adapt to lower, or higher, potential yields [13].

Detecting trends in fish stock productivity in a timely manner is difficult because short-term variability in recruitment and noisy abundance data usually combine to obscure changes in mean productivity of individual stocks [14,15]. Although temporal shifts in stock productivity tend to correspond to changes in environmental conditions [5,9], identifying the exact mechanisms driving the trends is often difficult because of a poor understanding of how marine fish stocks respond to environmental change [16,17]. However, insights into the dynamics of fish stock productivity can be gained by identifying common trends across multiple stocks [18,19]. In particular, identifying spatial patterns in productivity can help differentiate between alternative hypotheses used to explain the temporal trends [20]. For example, synchronous trends in productivity across geographically distinct stocks may suggest a common driver (e.g., regional or large-scale climate processes), whereas asynchronous changes in productivity across stocks may suggest a more localized driver (e.g., density-dependent effects).

For sockeye salmon, multi-stock analyses have indicated widespread declines in productivity over the past two decades for stocks ranging from Washington (WA) to Southeast Alaska [6]. These observed declines in productivity likely originate from a common oceanographic driver because sockeye salmon stocks throughout the southern part of their North American range show similar magnitude and directional changes in productivity [6]. Across broad spatial scales, productivity of sockeye, pink (*O*. *gorbuscha*), and chum salmon (*O*. *keta*) tend to be influenced similarly by changes in ocean conditions (e.g., sea surface temperature), suggesting that productivity of pink and chum salmon stocks in WA and British Columbia (BC) may have also declined over the past two decades [21]. However, productivity series between sockeye and chum salmon and sockeye and pink salmon tend to only be weakly to moderately correlated [22], possibly due to differences in life history strategies among species [23].

In this study, we asked whether productivity of wild North American pink and chum salmon stocks has declined over the past two decades and if so what is the spatial extent of the declines. In particular, our objectives were (1) quantify temporal trends in productivity for pink and chum salmon stocks throughout their North American range to determine the magnitude and direction of recent temporal shifts, and (2) investigate the extent of spatial heterogeneity in productivity trends to better understand the mechanisms driving temporal trends. To accomplish these objectives, we first estimated stock productivity trends for each of 99 pink and chum salmon stocks using a modified stock-recruit model and Kalman filter fitting procedure. We then estimated common productivity trends across all stocks using dynamic factor analysis (DFA). Together, this two-step modeling approach allowed us to quantify and summarize temporal trends in pink and chum salmon productivity across local, regional, and continental scales.

## Methods

### Salmon data

**Fig 1:**
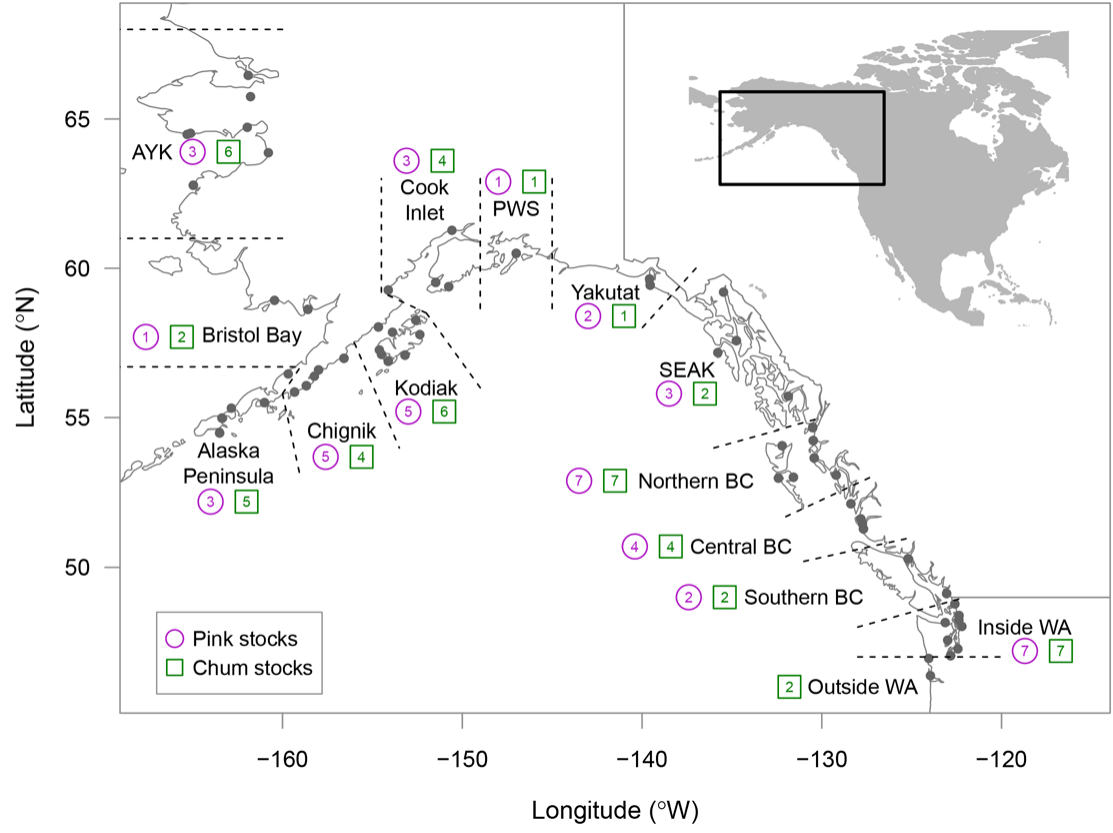
Study area showing the unique ocean entry locations for each salmon stock (solid dots). The 14 geographical regions are separated by dotted lines and the number of pink salmon and chum salmon stocks within a region are given by the numbers inside the circles (pink salmon) and squares (chum salmon). Map data from http://www.naturalearthdata.com.

We used wild spawner abundance and total recruitment (catch plus escapement) data for 53 chum salmon stocks (S1 Table) and 46 pink salmon stocks (S2 Table) from WA, BC, and Alaska (AK), to estimate indices of salmon productivity (Fig 1). Hatchery production was excluded. Data sets were, on average, 33 years long for chum salmon (range = 12–50 years) and 34 years for pink salmon (range = 15–56 years). Compared with stock-recruit data sets published and analyzed in Dorner et al. [12], which ended in brood year 1996 for pink salmon and 1995 for chum salmon, the updated data sets analyzed here represent a 20% (pink salmon) and 22% (chum salmon) increase in the average length of the time series. We organized the data sets into 14 geographic regions (Fig 1) to facilitate comparing productivity trends at the regional spatial scale. Data sets were aggregated across several populations in a few cases to ensure that catches were attributed to the correct spawning stocks. Where possible, aggregation was based on jurisdictional management units (see footnotes in S1 and S2 Tables).

Out of the 53 chum salmon data sets, 36 did not have full age-structured data. Without age-structure information, total returns in a given year cannot be properly allocated to a particular brood year of spawners. Therefore, to estimate total recruitment, we used assumptions about the age-structure of returning adults. Following Pyper et al. [24], we used two different age assumptions depending on data availability of a stock. If a stock had age-structured data for more than half of the total available brood years (4 stocks), we used the average age proportions to estimate recruitment for years without age data. If a stock had age data for less than half of the available brood years (32 stocks), we assumed all returns were age-4 for years without complete age information.

We chose to assume all returns were age-4 when little age data were available based on four lines of evidence that suggested this was an appropriate assumption. First, the dominant age of returning adults across the 17 stocks with full age data was age-4 (62% of all returns), with most chum salmon returning as age-3 and age-4 for WA and BC stocks (93% of all returns) and most salmon returning as age-4 and age-5 for Alaska stocks (86% of all returns). Second, recruitment series reconstructed using an age-4 assumption are strongly and positively correlated with observed recruitment series. For example, the average correlation between recruitment estimates calculated from observed age data and estimates reconstructed assuming all returns were age-4 across each of the 17 stocks with known age data was 0.82 (S1 Fig). Third, using a simulation model, Pyper et al. [24] found that for chum salmon, assuming all returns were age-4 resulted in less spurious autocorrelation in productivity estimates than using an average age assumption when fewer than half of the brood years had available age data. Fourth, the proportion of adults returning at particular ages is only moderately correlated for geographically close stocks, indicating that using age data from a nearby stock to fill in missing age data may not be appropriate. For instance, across the 17 stocks with known age data, the average correlation of age-4 return proportions between a given stock and the geographically closest stock with full age data was only 0.42.

### Productivity indices

We used two methods to estimate stock-specific time series of productivity for the 99 pink and chum salmon stocks. The first set of productivity indices were the residuals from a stationary Ricker spawner-recruit model, whereas the second set were time-varying Ricker *α* parameter values estimated using a Kalman filter procedure.

#### Stationary spawner-recruit models

We used residuals from a stationary Ricker spawner-recruit model as estimates of annual stock productivity, which removed potential within-stock density-dependent effects [21,25,26]. For each stock, the Ricker model was of the form,

$$log_{e}\left(R_{t}/S_{t}\right)=\alpha +\beta S_{t}+e_{t},$$ (1)

where *S*_*t*_ is the number of spawners in brood year *t*, *R*_*t*_ is the number of recruits resulting from *S*_*t*_, *α* is (constant) productivity at low spawner densities, *β* is the density-dependent effect, and *e*_*t*_ is the residual error assumed to be normally distributed with 0 mean and variance $\sigma_{e}^{2}$. The residuals from this model, standardized to a mean of 0 and a standard deviation of 1, were used as our first stock-specific productivity index.

#### Kalman filter spawner-recruit models

We estimated time-varying Ricker *α* parameter values for each stock using a Kalman filter procedure [27,28]. The primary benefit of using a Kalman filter to estimate stock productivity, rather than a stationary spawner-recruit model, is that the Kalman filter procedure allows the productivity parameter to vary non-linearly over time, rather than assuming a constant value [29]. The state-space Kalman filter model consisted of an observation equation describing the relationship between spawners and recruits and a process equation describing the unobserved state of nature (i.e., the *α* parameter) [30]. The observation equation was a Ricker model with a time-varying *α* parameter, i.e.,

$$log_{e}\left(R_{t}/S_{t}\right)=\alpha_{t}+\beta S_{t}+v_{t},$$ (2)

where *R*_*t*_ is the number of recruits in brood year *t*, *S*_*t*_ is the number of spawners, *α*_*t*_ is the annual stock productivity at low spawner abundances, *β* is the density dependence coefficient, and *v*_*t*_ is the observation error assumed to be normally distributed with a mean 0 and variance $\sigma_{v}^{2}$. The process equation was a random walk for the *α*_*t*_ parameter,

$$\alpha_{t}=\alpha_{t-1}+w_{t},$$ (3)

where *w*_*t*_ is the process error assumed to be normally distributed with mean 0 and variance $\sigma_{w}^{2}$. We chose a random walk because the underlying pattern of variation in productivity is unknown and a random walk allows for a wide variety of potential trends [31].

The Kalman filter model estimated annual values for the *α*_*t*_ parameter using an iterative Bayesian updating procedure whereby the prior value for *α*_*t*_ (i.e., *a*_*t*−1_) was updated based on how well *a*_*t*−1_ predicted the following year’s *log*_*e*_(*R*_*t*_/*S*_*t*_) [28]. This procedure produced a time series for the *α* parameter where each *α*_*t*_ value only depended on data up through year *t*. Following Peterman et al. [29], we then applied a fixed interval smoother to the time series of *α*_*t*_ parameters. Unlike the Kalman filter procedure, which estimated the earliest *α*_*t*_ value first and moved forward through time, the smoothing procedure worked backwards starting with the most recent filtered *α*_*t*_ value [32]. For each *α*_*t*_ value, the smoother calculated a weighted average between the estimate of the filtered value at time *t* and the smoothed estimate at time *t* + 1, thereby producing estimates of *α*_*t*_ that were based on the whole time series, rather than only the previously estimated values. This smoothed time series of *α*_*t*_, standardized to a mean of 0 and a standard deviation of 1, was used as our second stock-specific productivity index.

The other parameters of the Kalman filter model, (i.e., $\beta ,\;\sigma_{v}^{2}$, and $\sigma_{w}^{2}$) were assumed constant over time and were estimated using maximum likelihood methods. Details of the estimation procedure are described in Peterman et al. [31] and Peterman et al. [29]. An S-PLUS implementation of the Kalman filter procedure used here is available in Dorner et al. [12].

To further characterize the Kalman filter models, we also calculated the signal-to-noise ratio ($\sigma_{w}^{2}\;/\;\sigma_{v}^{2}$) for each stock to quantify the variance partitioned to the temporal trend compared with the high-frequency variation (i.e., noise). We also calculated average signal-to-noise ratios for each region and species (average $\sigma_{w}^{2}$ / average $\sigma_{v}^{2}$).

### Common productivity trends

To more clearly identify shared trends in productivity, we used DFA models, which are a class of multivariate autoregressive state-space models, to estimate common productivity trends across all stocks within a species. Dynamic factor analysis aims to estimate a specific number of common trends (*M*) given a set of multivariate time series, which unlike other time series analyses (e.g., ARIMA models), can be used to analyze short, non-stationary time series with missing values [33,34]. For the DFA models used in this paper, the individual stock productivity time series were modeled as a function of (1) a linear combination of the specified number of trends, and (2) a noise term. Following Zuur et al. [33], the DFA model can be written in matrix form as,

$$\begin{array}{l} y_{t}=Z\tau_{t}+e_{t}\\ \tau_{t}=\tau_{t-1}+f_{t}, \end{array}$$ (4)

where **y**_*t*_ is the vector of observed data at time *t*, **τ**_*t*_ is an *M* x 1 vector of common trends, **Z** is the stock-specific factor loadings for each trend, which describe the form of the linear combination of trends, **e**_*t*_ is the observation error term assumed to be normally distributed with mean 0 and variance-covariance matrix **R**, and **f**_*t*_ is the process error also assumed to be normally distributed with mean 0 and variance-covariance matrix **Q**. In our study, all DFA trends were modeled using a random walk and were estimated using a Kalman filter/smoothing algorithm, which restricted the trends to smooth curves [34].

We fit a total of 24 DFA models for each species using different combinations of trends and observation error variance-covariance matrices. All DFA models were fit using the residuals from the stationary Ricker models, rather than the Kalman filter productivity indices, because the DFA model already includes a Kalman filtering procedure. All productivity series were truncated to the period after 1979 because many stocks had considerable missing data or no data prior to 1980 (e.g., most stocks in BC). We fit models with 1–6 common trends and four different observation error variance-covariance matrices (i.e., **R**) including (1) a diagonal and equal variance-covariance matrix where all stock variances were equal and covariances between stocks were assumed 0, (2) a diagonal and unequal matrix where the variances were allowed to vary between stocks and covariances were assumed 0, (3) an equal matrix where the variances were all equal and the covariances were allowed to be non-zero but were all equal, and (4) a north-south matrix, which was similar to the equal variance-covariance matrix but variances and covariances differed for northern stocks (AK) and southern stocks (BC and WA) separately.

We used the small-sample Akaike Information Criterion (AIC_C_) as a model selection criterion [35,36]. AIC_C_ values were evaluated based on four criteria: (1) the most parsimonious model was defined as the model with the lowest AIC_C_ value, (2) equally plausible models were defined as models with AIC_C_ values within 3 AIC_C_ units of the lowest, (3) less plausible models had AIC_C_ values greater than 3 from the minimum and were rejected with caution, and (4) models with AIC_C_ values greater than 10 compared to the minimum were rejected with confidence [36]. To further evaluate the DFA model fits, we also examined model residuals, fitted values, estimated trends, and stock loadings for each model. All models were fit using maximum likelihood in R using the MARSS package [37,38].

### Spatial covariation

We quantified spatial covariation in productivity series within and across geographic regions by calculating Pearson correlation coefficients for each unique pair of salmon stocks within a species. We estimated between-stock covariation using both the residual and Kalman filter *α*_*t*_ productivity indices and only computed correlation coefficients between two stocks if the stocks had at least 10 years of overlap. Because previous research has indicated that the strength of spatial synchrony in productivity may change over time [6,39], we calculated pairwise correlations for three time periods: (1) all available brood years, (2) earliest brood year–1990, and (3) 1991–last brood year. For the early and late periods, we restricted the analysis to only the residual productivity index because of potential difficulties in fitting the Kalman filter models with such short time series.

### Sensitivity analysis

We initially assumed that because even and odd year pink salmon runs occupy the same physical habitat in alternate years that even and odd year runs could be treated as a single entity. However, pink salmon have a fixed 2-year life cycle where even and odd year brood lines are reproductively isolated. To check the sensitivity of our results to our assumption that even and odd year brood lines could be combined, we repeated the Kalman filter, DFA, and covariation analyses separately for even and odd year pink salmon brood lines.

## Results

### Individual stock productivity trends

**Fig 2:**
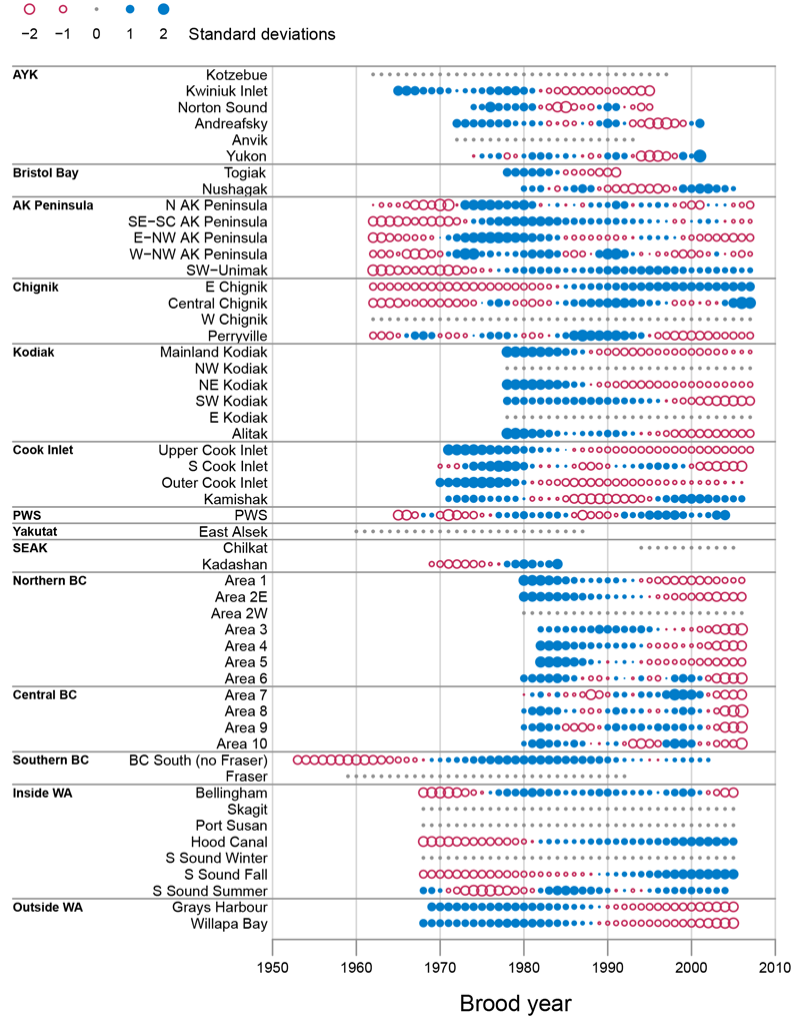
Chum salmon productivity time series from the Kalman filter models. Each time series shows the smoothed *α*_*t*_ estimates from the Kalman filter model where each series is standardized to a mean of 0 and standard deviation of 1. The ordinate gives the stock, which are arranged south (bottom) to north (top) and grouped by geographic region. The area of the circle indicates the magnitude of the productivity values. Open circles represent negative values (red) and filled circles indicate positive values (blue). Small solid grey dots indicate zero values, whereas missing values are not shown.

Time series of the Kalman filter reconstructed *α*_*t*_ values indicated widespread declines in chum salmon productivity since at least brood year 2000 (Fig 2). The strongest and most consistent declines in chum salmon productivity were for stocks in WA and BC with 81% of southern chum salmon stocks with non-constant *α*_*t*_ series showing recent declines in productivity. Among the stocks with recent declines in productivity, two qualitative trends were observed. The first trend, exemplified by stocks in the Outside WA and Northern BC regions, was characterized by gradual, but consistent declines in productivity since the mid 1980s (Fig 2). The second trend was characterized by an abrupt and steep decline in productivity starting around brood year 2000 and was observed for the four Central BC stocks, as well as the northern most Inside WA stock and the southern most Northern BC stock. In contrast to the widespread declines in productivity for WA and BC chum salmon stocks, three Inside WA stocks showed increasing productivity trends in recent years. For AK chum salmon stocks, there was weaker evidence for widespread declines in productivity with 67% of stocks showing recent declines in productivity. Among the AK regions, chum salmon stocks in the Kodiak, and to a lesser extent the AK Peninsula and Cook Inlet regions showed the most consistent declines in productivity in recent years (Fig 2).

**Fig 3:**
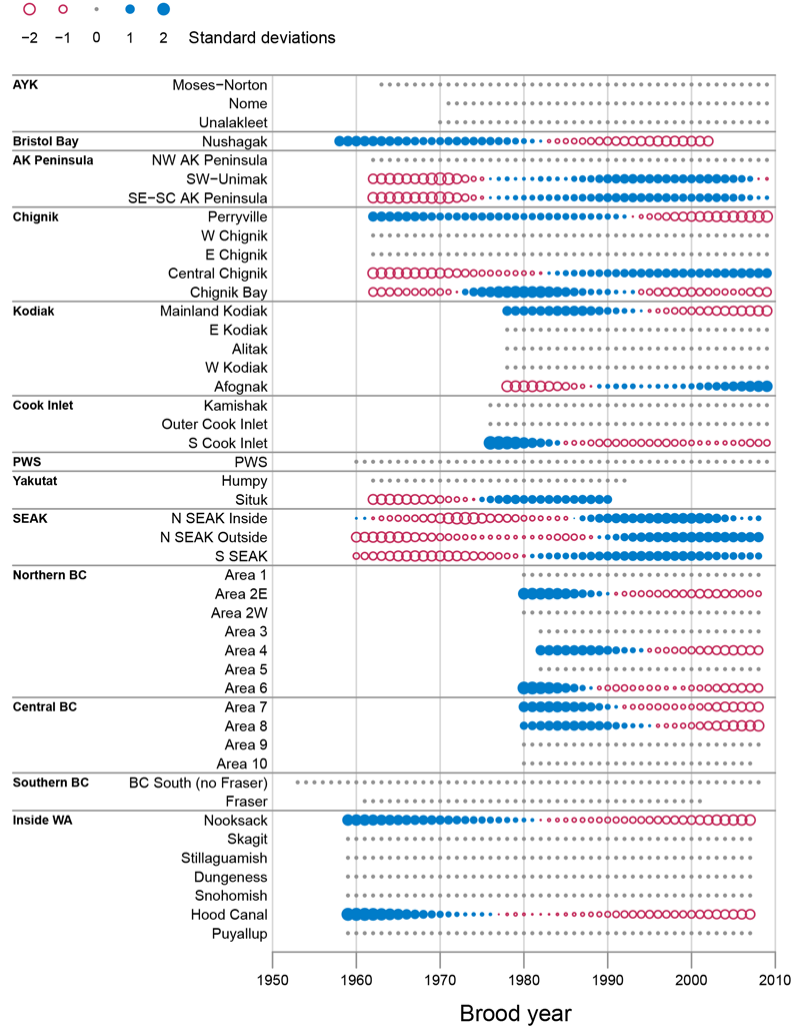
Pink salmon productivity time series from the Kalman filter models. Each time series shows the smoothed *α*_*t*_ estimates from the Kalman filter model where each series is standardized to a mean of 0 and standard deviation of 1. The ordinate gives the stock, which are arranged south (bottom) to north (top) and grouped by geographic region. The area of the circle indicates the magnitude of the productivity values. Open circles represent negative values (red) and filled circles indicate positive values (blue). Small solid grey dots indicate zero values, whereas missing values are not shown.

For pink salmon, the Kalman filter *α*_*t*_ series indicated a general pattern of declining productivity for WA and BC stocks and a more variable pattern for AK stocks (Fig 3). All stocks in the three southern regions (i.e., Inside WA, Central BC, and Northern BC) with non-constant *α*_*t*_ series showed declining or below average productivity values since at least brood year 1990 (Fig 3). In contrast, there was less consist patterns in AK pink salmon stocks with several regions having stocks with both increasing and decreasing productivity trends in recent years (e.g., Kodiak and Chignik). Across all AK pink salmon stocks with non-constant *α*_*t*_ series, 64% had increasing or above average productivity in recent years. However, only the Southeast Alaska and AK Peninsula regions had stocks that showed consistent increasing productivity trends in recent years with all five stocks with non-constant *α*_*t*_ values having above average productivity since at least brood year 1990 (Fig 3).

The Kalman filter *α*_*t*_ series for pink salmon had many more constant series (57% of pink salmon stocks) compared to chum salmon (23% of stocks; Figs 2 and 3). These constant series result from the Kalman filter partitioning the variability as high-frequency variation (i.e., noise) not related to an underlying productivity trend (i.e., signal). This difference between the strength of the underlying signal compared with high-frequency noise was also evident in the considerably higher average signal-to-noise ratio for chum salmon (0.23) compared to pink salmon (0.01; Table 1), suggesting that the low-frequency trend is a less important part of the total variation for pink salmon than for chum salmon.

**Table 1:** Average regional signal-to-noise ratio for Kalman filter Ricker models.

Region	Chum	Pink
Outside WA	0.005	-
Inside WA	0.046	0.008
Southern BC	0.014	0.000
Central BC	1.566	0.010
Northern BC	0.188	0.012
Southeast Alaska	0.389	0.068
Yakutat	0.000	0.017
Prince William Sound	>10	0.000
Cook Inlet	0.160	0.013
Kodiak	0.032	0.003
Chignik	0.117	0.034
Alaska Peninsula	0.589	0.041
Bristol Bay	0.331	0.015
AYK^a^	0.573	0.000

^a^ Arctic Yukon Kuskokwim.

The residual productivity series had considerably more short-term high-frequency variability than the reconstructed Kalman filter *α*_*t*_ series, making patterns harder to discern (S2 and S3 Figs). Despite the high amount of inter-annual noise, the general patterns observed in the Kalman filter *α*_*t*_ series were also observed in the residual series. For chum salmon, the residual series also indicated widespread declines in productivity for WA and BC chum salmon stock, although the recent trends tended to be more similar to those of the Northern BC *α*_*t*_ series with steep declines in productivity since brood year 2000 (S2 Fig). For pink salmon, clear patterns in residual series were not observed for most stocks, which corresponds with the low signal-to-noise ratios observed for pink salmon *α*_*t*_ estimates (S3 Fig).

### Common productivity trends

**Fig 4:**
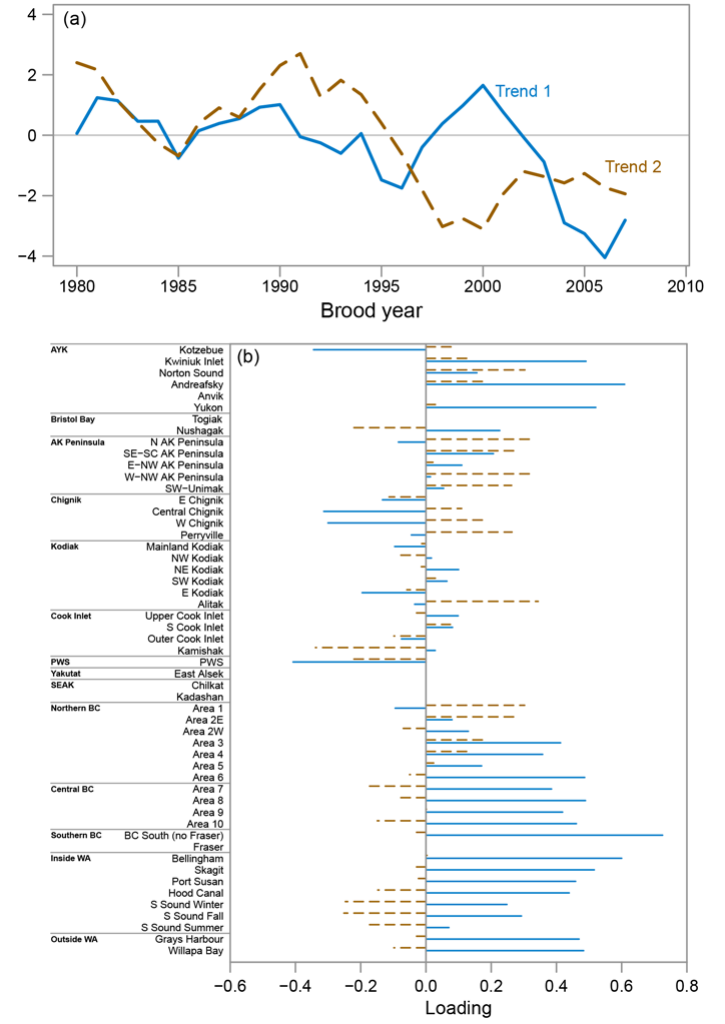
Chum salmon common productivity trends from the best fit dynamic factor analysis model. (a) The first (solid line) and second (dashed line) common productivity trends and (b) stock specific loadings for the first (solid horizontal bars) and second (dashed horizontal bars) common trends. In the bottom panel, stocks are ordered south (bottom) to north (top) and are grouped by geographic region.

For chum salmon, there was strong support from the data for a DFA model with two common trends and the north-south variance-covariance matrix (Table 2). The first common trend for chum salmon was characterized by fairly constant productivity from 1980–2000 and then a steep decline in productivity from 2000–2007 (solid line in Fig 4A). In contrast, the second common productivity trend was characterized by an increase in productivity from 1985–1991 and then a decline in productivity from 1991–1998 and low productivity since 1998 (dashed line in Fig 4A). WA and BC stocks tended to have high positive loadings on the first trend (average loading for WA and BC stocks = 0.36) and weaker negative loadings on the second trend (average loading = -0.03), whereas AK stocks had more variable loadings between the two trends with no clear patterns within or across regions (Fig 4B).

**Table 2:** Summary of top three dynamic factor analysis models for chum and pink salmon separately.

Species	N	Parameters	Trends	Var-covar	Log-likelihood	AICc	*Δ*AICc
Chum	1213	98	2	north-south	-1508.8	3230.9	0.0
	1213	52	1	north-south	-1563.8	3236.3	5.3
	1213	95	2	equal-var-covar	-1519.2	3244.7	13.7
Pink	1075	47	1	north-south	-1392.4	2883.3	0.0
	1075	88	2	north-south	-1365.6	2923.2	39.9
	1075	44	1	equal-var-covar	-1418.3	2928.4	45.2

N gives the number of data points included in the model; Parameters gives the total number of parameters in the model; Trends gives the number of trends the model was fit with; Var-covar gives the variance-covariance matrix structure used to fit the model where north-south indicates variances and covariances were equal across stocks but were allow to differ between northern (i.e., AK) and southern (i.e., WA and BC) stocks and equal-var-covar indicates variances and covariances were equal for all stocks.

**Fig 5:**
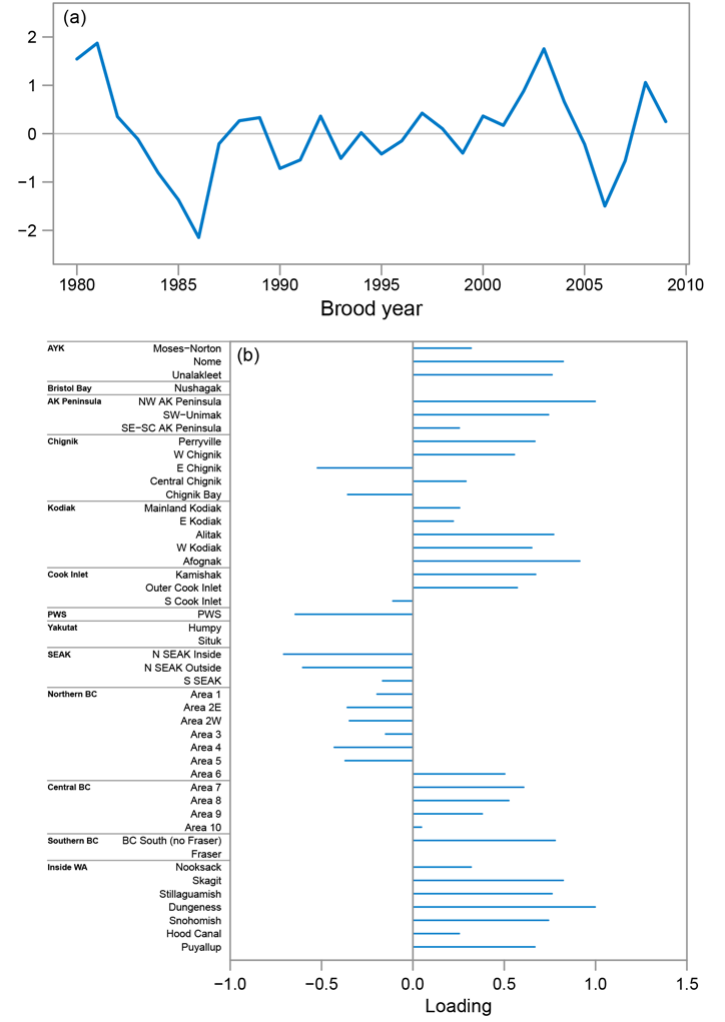
Pink salmon common productivity trend from the best fit dynamic factor analysis model. (a) the single pink salmon common trend and (b) the stock specific loadings for the single common trend. In the bottom panel, stocks are ordered south (bottom) to north (top) and are grouped by geographic region.

The pink salmon DFA model with the lowest AIC_C_ value included a single common trend and the north-south variance-covariance matrix (Table 2). Unlike chum salmon, the common trend for pink salmon did not indicate declines in productivity in recent years (Fig 5A). The single common pink salmon trend was characterized by a steep decline in productivity from 1981 to 1986 followed by fairly constant productivity that varied around a constant mean value for the remainder of the series (Fig 5A). Stock loadings on this trend oscillated moving south to north with stocks south of Northern BC and north and west of Prince William Sound tending to have positive loadings on the trend and stocks ranging from Northern BC to Prince William Sound tending to have negative loadings (Fig 5B).

### Spatial covariation

**Fig 6:**
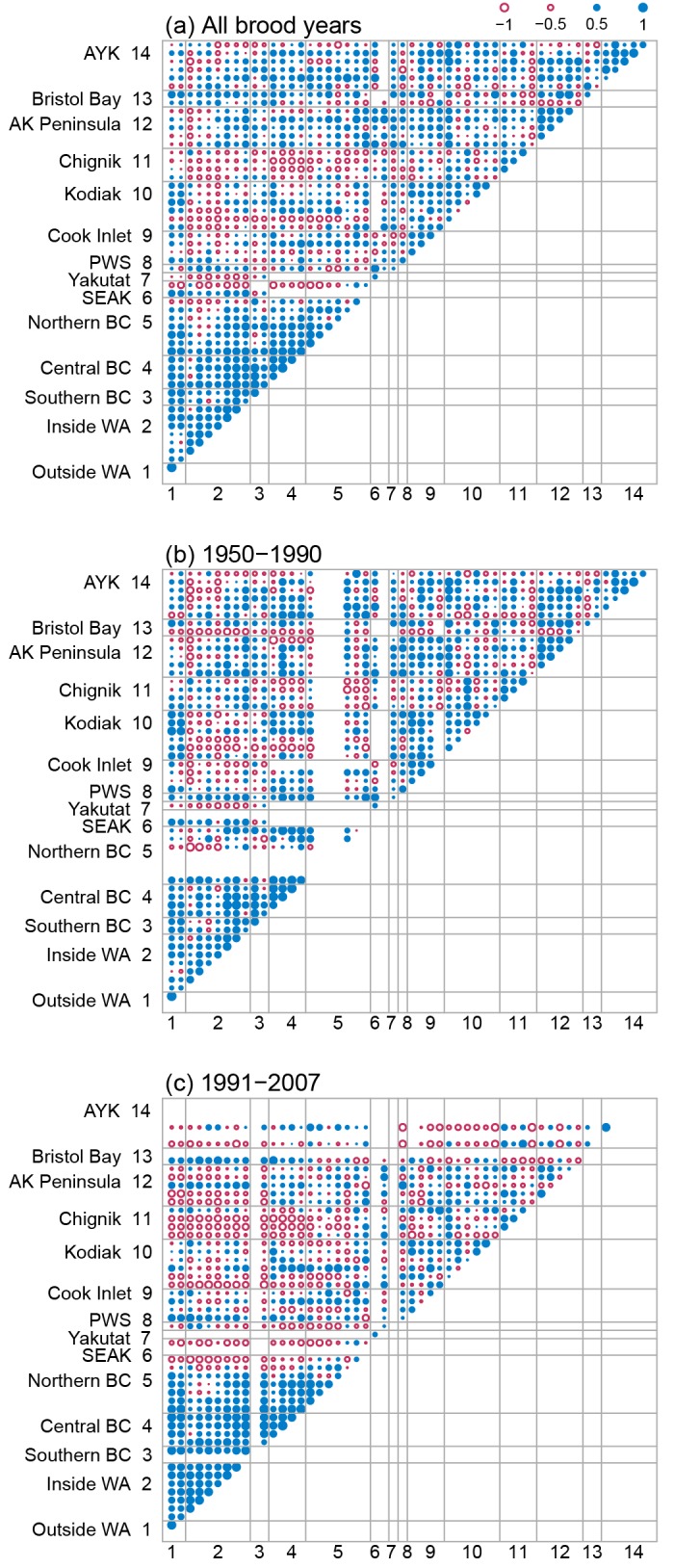
Pairwise correlation coefficients between chum salmon stock residual productivity time series for three time period. (a) between-stock correlation coefficients calculated using all available brood years, (b) correlations for brood years 1950–1990, and (c) correlations for brood years 1991–2007. The magnitude of the between-stock correlation is given by the area of the circle with larger circles representing larger correlations than smaller circles. Negative correlations between stocks are shown as open circles (red) and positive correlations are shown as filled circles (blue). Stocks are grouped by geographic region, which are arranged south (bottom, left) to north (top, right).

**Fig 7:**
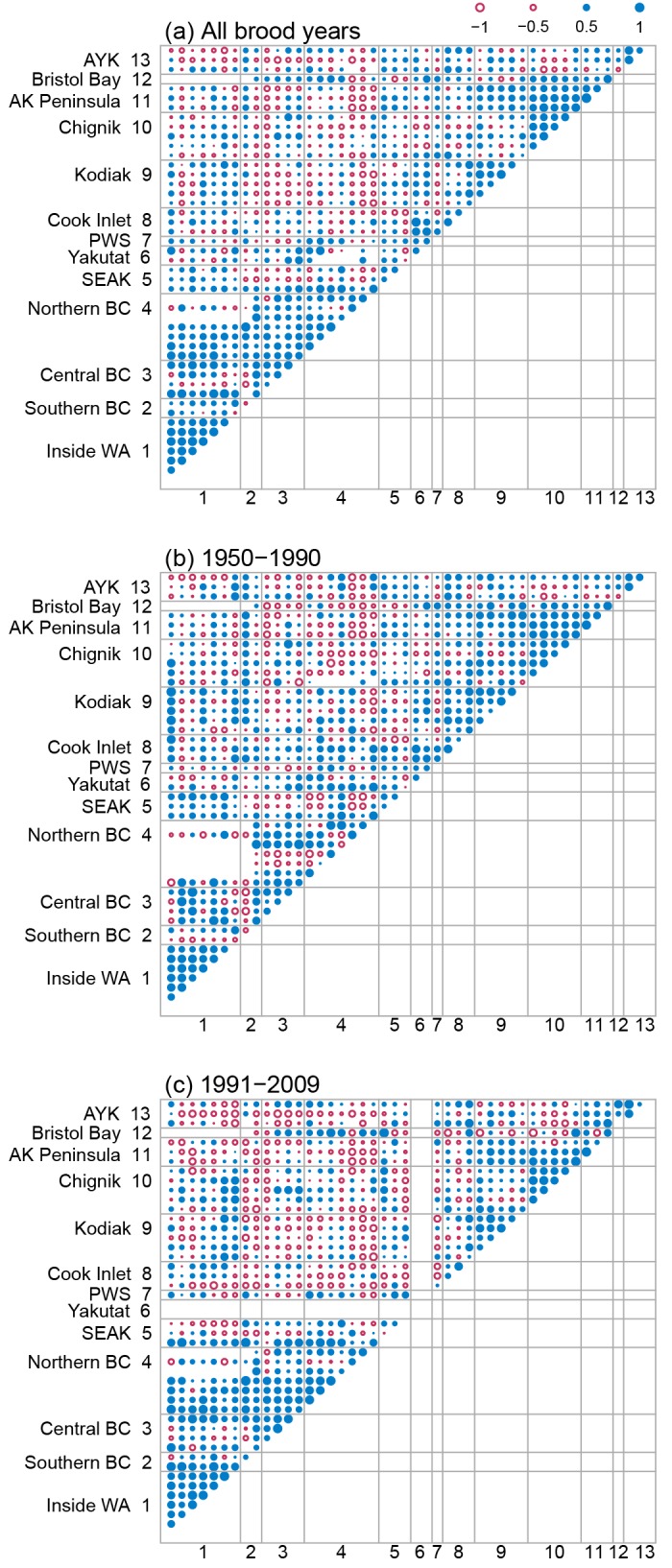
Pairwise correlation coefficients between pink salmon stock residual productivity time series for three time period. (a) between-stock correlation coefficients calculated using all available brood years, (b) correlations for brood years 1950–1990, and (c) correlations for brood years 1991–2009. The magnitude of the between-stock correlation is given by the area of the circle with larger circles representing larger correlations than smaller circles. Negative correlations between stocks are shown as open circles (red) and positive correlations are shown as filled circles (blue). Stocks are grouped by geographic region, which are arranged south (bottom, left) to north (top, right).

Productivity indices for both chum and pink salmon stocks within a region were, on average, strongly and positively correlated (Table 3; diagonal boxes in Figs 6 and 7; average within-region correlation = 0.66 and 0.67 for chum and pink salmon respectively), suggesting there are synchronous trends in productivity within regions. There was little evidence that the magnitude of within-region covariation changed over time for either pink or chum salmon, with the average within-region correlation for the early period (chum $\bar{r}$ = 0.67; pink $\bar{r}$ = 0.67; Figs 6B and 7B) being nearly equal to the late period (chum $\bar{r}$ = 0.63; pink $\bar{r}$ = 0.66; Figs 6C and 7C). Across all brood years and regions, positive covariation was strongest for chum and pink salmon stocks in WA and BC with little evidence for positive covariation between distant regions (Figs 6A and 7A). Positive covariation between stocks for both chum and pink salmon in WA and BC was twice as strong in the most recent period (1991–2007; chum $\bar{r}$ = 0.41; pink $\bar{r}$ = 0.29) compared to the early period (1950–1990; chum $\bar{r}$ = 0.23; pink $\bar{r}$ = 0.12). Similarly, negative covariation between northern and southern salmon stocks was more pronounced in the most recent period compared to the early period (Figs 6 and 7).

**Table 3:** Average correlations between unique pairs of stocks within geographic regions.

	Ricker		Kalman	
Region	Chum	Pink	Chum	Pink
Outside WA	0.96	-	0.99	-
Inside WA	0.58	0.68	0.43	0.81
Southern BC	0.87	0.63	0.74	0.36
Central BC	0.79	0.64	0.81	0.90
Northern BC	0.47	0.50	0.65	0.69
Cook Inlet	0.62	0.68	0.68	0.53
Kodiak	0.46	0.62	0.46	0.44
Chignik	0.59	0.66	0.36	0.40
Alaska Peninsula	0.64	0.80	0.68	0.91
Bristol Bay	0.74	-	0.81	-
AYK^a^	0.57	0.77	0.46	0.49

Average correlations are shown for both the Ricker residual and Kalman filter *α*_*t*_ productivity series for each geographic region. Average correlations were only calculated if at least two stocks had sufficient data within a region, therefore, some regions (e.g., Prince William Sound, Southeast Alaska, and Yakutat) are not shown or have no values.

^a^ Arctic Yukon Kuskokwim.

Covariation patterns for the Kalman filter *α*_*t*_ productivity indices were similar to those of the residual series including (1) positive covariation between stocks within a region, (2) positive covariation between stocks in different regions in WA and BC, although this pattern was not as strong for chum salmon *α*_*t*_ series, and (3) weak covariation between stocks that enter the ocean at distant locations (Table 3; S4 and S5 Figs).

### Sensitivity analysis

Our results were not sensitive to our assumption that even and odd year pink salmon brood lines could be treated as a single entity. In particular, Kalman filter *α*_*t*_ series for stocks with even and odd year runs tended to show similar trends (S6 Fig), and with the exception of the even year Area 6 stocks, all WA and BC pink salmon stocks with non-constant *α*_*t*_ series showed recent declines in productivity, similar to the trends observed when odd and even years brood lines were combined (S6 Fig). Both even and odd year DFA models with the lowest AIC_C_ values included a single common trend and the north-south variance-covariance matrix, which corresponds to the DFA model selection results for the combined pink salmon analysis. The common trends for even and odd year brood lines showed similar patterns to the combined DFA analysis with no evidence for strong declines in productivity in recent years (S7 Fig). Spatial covariation patterns were also similar between the even and odd year brood lines and the combined pink salmon analysis, including strong positive covariation between stocks in the same region, positive covariation between stocks in WA and BC, and weak covariation between distant stocks (S8 Fig).

## Discussion

Our results provide evidence that productivity of wild chum salmon stocks, and to a lesser extent wild pink salmon stocks, has declined over the past two decades throughout WA and BC. Specifically, (1) productivity for the majority of chum salmon stocks in WA and BC has declined over the past two decades, although the functional form of declines varied across regions with Central BC stocks showing an abrupt and steep decline in productivity starting around brood year 2000 and stocks in the Outside WA and Northern BC regions showing a more gradual declining trend, (2) trends in productivity for AK pink and chum salmon stocks were more variable with some regions and stocks showing declines in productivity and others showing increases, (3) there was strong positive covariation of productivity series within regions, and (4) covariation between productivity series for stocks in WA and BC has become stronger in recent years for both pink and chum salmon. In general, our results suggest that productivity of pink and chum salmon stocks is driven by common processes operating at the regional or multi-regional spatial scale, and the effects of these drivers may not be constant through time.

Evidence for widespread declines in productivity was stronger for chum salmon than for pink salmon. With the exception of the Kalman filter results for Inside WA chum salmon stocks, both the DFA and Kalman filter analyses indicated that chum salmon stocks throughout WA and BC have experienced declines in productivity over the past two decades. In contrast, the Kalman filter analysis for pink salmon indicated that the low frequency signal (i.e., trend) tended to contribute less to the total variability for many stocks in WA and BC. This corresponded with the single common DFA trend for pink salmon that did not show strong increasing or decreasing trends in productivity over the past two decades. However, across the WA and BC pink salmon stocks where a trend in productivity was observed, the trend was consistently downward over the past two decades. This finding that most chum and some pink salmon stock productivities have declined markedly throughout WA and BC is consistent with the findings of Peterman and Dorner [6] that indicated widespread declines in productivity for sockeye salmon stocks throughout WA and BC. This general correspondence of productivity trends across species is not surprising as all three species use similar marine environments [40], have considerable overlap in marine diets [41], and have been shown to respond similarly to changes in oceanographic conditions [21].

Despite the general coherence in productivity trends across species, there were some important differences between our pink and chum salmon productivity trends and those previously reported for sockeye salmon. In particular, Peterman and Dorner [6] indicated that sockeye salmon declines in productivity extended northward into Southeast Alaska. We did not have recent chum salmon data for Southeast Alaska stocks, however, we found little evidence for declines in pink salmon productivity in Southeast Alaska. Instead, the Kalman filter *α*_*t*_ series indicated above average or increasing productivity trends for all three Southeast Alaska pink salmon stocks since at least brood year 1990 (Fig 3). While it is not clear what is driving this difference in trends between pink and sockeye salmon in Southeast Alaska, it may be because of differences in how different salmon species use freshwater and marine habitats. For example, sockeye salmon tend to rear in freshwater lakes for 1–2 years, whereas pink salmon migrate to the ocean soon after emergence [23]. The additional residence time in freshwater for sockeye results in sockeye tending to be larger at the time of ocean entry compared to pink salmon, which may influence mortality rates during the early marine residence period [42–44].

We observed a general increasing trend in productivity for chum salmon stocks in the Inside WA region, which was opposite of the declining productivity trends observed for chum salmon stocks in the four other geographic regions in WA and BC. More specifically, the South Sound and Hood Canal chum salmon stocks had increasing trends in the Kalman filter *α*_*t*_ series and weak covariation with other WA and BC chum salmon stocks, particularly during the 1950–1990 period. Surprisingly, these southern Puget Sound stocks also differed from chum salmon stocks that enter the ocean in northern Puget Sound (e.g., the Bellingham stock). One potential explanation for why productivity trends of southern Puget Sound chum salmon stocks are different is differences in physical and biological conditions in different parts of Puget Sound. For example, Duffy et al. [45] indicated that in 2001 and 2002, sampling sites in southern Puget Sound had consistently higher sea surface temperatures and lower salinity values compared to northern Puget Sound sampling sites. Similarly, Duffy et al. [46] indicated that diets of chinook salmon differed between northern and southern Puget Sound with fish sampled in southern Puget Sound tending to have higher stomach fullness compared to fish sampled in northern Puget Sound. Another potential explanation for changes in productivity of Puget Sound area salmon stocks is concerted freshwater and estuarine habitat restoration efforts including changes in water use and land use practices [47], improvements to freshwater barriers such as culverts [48], and changes in shoreline modifications in the estuarine environment [49,50]. These localized differences within Puget Sound suggests that both environmental and habitat conditions in this area may be leading causes of the observed differences.

Similar to Peterman and Dorner [6] and Dorner et al. [12], we found strong positive covariation in productivity series between pink and chum salmon stocks within the same region and between stocks in adjacent regions. This finding also agrees with several previous studies that showed productivity series of North American pink, chum, sockeye, chinook, and coho salmon all tend to exhibit strong spatial synchrony at the scale of 100 to 1000 km [39,51–53]. This regional-scale spatial synchrony suggests that Pacific salmon productivity is largely driven by a common driver or drivers that operate at the regional or multi-regional spatial scale. Mechanisms that operate over these spatial scales may include freshwater or marine processes such as disease or pathogens [54], changes in stream flow and stream temperature [55], competition with abundant hatchery salmon [56], or shifts in oceanographic condition such as the timing of the spring phytoplankton bloom or sea surface temperature [21,52].

The observed patterns of covariation in productivity are also consistent with previously reported covariation patterns for wild pink and chum salmon abundances [57,58]. In particular, Stachura et al. [57] indicated that abundances of wild pink and chum salmon tend to have similar temporal trends across regions, which corresponds with our result that productivity tends to strongly covary between stocks in adjacent regions. In contrast, both Stachura et al. [57] and Ruggerone et al. [58] indicated a general pattern of increasing abundances of North American pink salmon and weak or no trends for chum salmon abundances over the past forty years. This differs from our finding that productivity of most chum and some pink salmon stocks has declined over a considerable portion of their geographic range. One possible explanation for this divergence in trends in productivity and abundance is that abundance estimates can be confounded with several factors including changes in harvest management, hatchery production, and spawner abundances.

Our results broadly agree with those of several previous studies that suggested spatial covariation between demographic rates of nearby sockeye and chinook salmon stocks have increased in recent decades [6,39,59]. In particular, our results indicated that covariation between southern area chum and pink salmon productivity series was higher during the 1991–2007 period than the 1950–1990 period. This agrees with the results of Peterman and Dorner [6], which indicated covariation between WA and BC sockeye salmon stocks was higher during the period 1995–2004 than during earlier periods. Similarly, our results are consistent with those of Kilduff et al. [39] that also indicated increased covariation of survival rates between chinook salmon stocks since the early 1990s. This increased covariation suggests that the mechanisms driving covariability between salmon stocks may have changed (e.g., become stronger) over recent years. Indeed, there is some evidence that relationships between environmental variation and salmon dynamics are not stationary, but instead change over time, as indicated by the frequent breakdown of correlations between environmental factors and stock demographic rates [16].

Regardless of the underlying mechanisms driving the trends, our results indicate that productivity of most chum salmon and some pink salmon stocks is non-stationary, which has important management and conservation implications if non-stationarity is not accounted for when setting management targets. Our results support the idea that the use of stationary spawner-recruit models to estimate stock productivity may be inappropriate for setting management targets, such as harvest rates or escapement goals, particularly in situations where large changes in productivity have occurred, such as those observed for chum salmon throughout BC [13,31]. Indeed, in cases where salmon productivity has abruptly declined, empirical analysis has indicated that failing to account for non-stationarity in the Ricker *α* parameter often leads to overestimates of the exploitation rate for a stock compared to using a Kalman filter fitting procedure [29]. This overestimation of the exploitation rate occurs because methods that assume stationarity in the Ricker *α* parameter put more weight on historical data compared to the Kalman filter model, which tends to update parameter estimates faster [31]. This outcome can also go in the opposite direction, that is, in situations where productivity has increased, potentially leading to lower economic benefit because of overly conservative harvest rates resulting from underestimating stock productivity [12,31].

In conclusion, our results indicate that the majority of North American chum and some pink salmon stocks have experienced large shifts in productivity over the past two decades, and at least for WA and BC stocks, these shifts have been downward. The coherence of these declines over regional and multi-regional spatial scales suggests that physical or biological factors operating at similar spatial scales are likely driving the observed trends. Because productivity is an important determinant of marine and anadromous fish stock dynamics, the sharp or gradual shifts in productivity observed here for pink and chum salmon can have important consequences for management and conservation. In particular, this research further demonstrates the need to account for time varying demographic rates in stock assessments to quickly detect temporal changes that may otherwise lead to an increased risk of overexploitation.

## Supporting Information

S1 FigChum salmon recruitment estimated using observed age proportions (blue lines) and estimated assuming all returns were age-4 (red lines) for the 17 stocks with full age data.Recruitment series are standardized to a mean of 0 and standard deviation of 1. Correlation coefficients between the two recruitment series are given within each panel (*r*) and $\bar{r}$ gives the average correlation across the 17 stocks.(PDF)

S2 Fig Chum salmon productivity time series of residuals from the stationary Ricker model. The residual series are standardized to a mean of 0 and standard deviation of 1. The ordinate gives the stock, which are arranged south (bottom) to north (top) and grouped by geographic region. The area of the circle indicates the magnitude of the productivity values. Open circles represent negative values (red) and filled circles indicate positive values (blue).
(PDF)

S3 Fig Pink salmon productivity time series of residuals from the stationary Ricker model. The residual series are standardized to a mean of 0 and standard deviation of 1. The ordinate gives the stock, which are arranged south (bottom) to north (top) and grouped by geographic region. The area of the circle indicates the magnitude of the productivity values. Open circles represent negative values (red) and filled circles indicate positive values (blue).
(PDF)

S4 Fig Pairwise correlation coefficients between chum salmon stock Kalman filter *α*_*t*_ productivity time series. Correlations were calculated using all available brood years. The magnitude of the correlation is given by the area of the circle with larger circles representing larger correlations than smaller circles. Negative correlations between stocks are shown as open circles (red) and positive correlations are shown as filled circles (blue). Stocks are grouped by geographic region, which are arranged south (bottom, left) to north (top, right).
(PDF)

S5 Fig Pairwise correlation coefficients between pink salmon Kalman filter *α*_*t*_ productivity time series. Correlations were calculated using all available brood years. The magnitude of the correlation is given by the area of the circle with larger circles representing larger correlations than smaller circles. Negative correlations between stocks are shown as open circles (red) and positive correlations are shown as filled circles (blue). Stocks are grouped by geographic region, which are arranged south (bottom, left) to north (top, right).
(PDF)

S6 Fig Pink salmon productivity time series from the Kalman filter models for even and odd year brood lines. Each time series shows the smoothed *α*_*t*_ estimates from the Kalman filter model where each series is standardized to a mean of 0 and standard deviation of 1. Even and odd year brood lines are shown concurrently for each stock with the even year brood lines offset slightly above the odd year brood lines. The ordinate gives the stock, which are arranged south (bottom) to north (top) and grouped by geographic region. The area of the circle indicates the magnitude of the productivity values. Open circles represent negative values (red) and filled circles indicate positive values (blue). Small solid grey dots indicate zero values, whereas missing values are not shown.
(PDF)

S7 Fig Pink salmon common productivity trends from the best fit dynamic factor analysis model for even and odd brood lines. (a) Even brood year (solid line) and odd brood year (dashed line) common productivity trends and (b) stock specific loadings for the even year (solid horizontal bars) and odd year (dashed horizontal bars) common trends. In the bottom panel, stocks are ordered south (bottom) to north (top) and are grouped by geographic region.
(PDF)

S8 Fig Pairwise correlation coefficients between pink salmon stock residual productivity time series for even and odd brood lines. (a) between stock correlation coefficients for odd year runs and (b) correlations for even year runs. The magnitude of the correlation is given by the area of the circle with larger circles representing larger correlations than smaller circles. Negative correlations between stocks are shown as open circles (red) and positive correlations are shown as filled circles (blue). Stocks are grouped by geographic region, which are arranged south (bottom, left) to north (top, right).
(PDF)

S1 Table Chum salmon data set summary. Brood years gives the range of years available for each stock; N gives the total brood years with data; R/S gives the average spawner to recruit ratio over all available brood years; Stationary *α* and *β* give the parameter estimates from the best fit stationary Ricker model; Kalman filter *α*_*t*_ gives the average *α*_*t*_ value and SD gives the standard deviation for the *α*_*t*_ series; Kalman filter S/N gives the signal-to-noise ratio for that stock.
(PDF)

S2 Table Pink salmon data set summary. Brood years gives the range of years available for each stock; N gives the total brood years with data; R/S gives the average spawner to recruit ratio over all available brood years; Stationary *α* and *β* give the parameter estimates from the best fit stationary Ricker model; Kalman filter *α*_*t*_ gives the average *α*_*t*_ value and SD gives the standard deviation for the *α*_*t*_ series; Kalman filter S/N gives the signal-to-ratio for that stock.
(PDF)
