# Supplementary figures and images for: Regional-Scale Declines in Productivity of Pink and Chum Salmon Stocks in Western North America
Source: PLoS One. 2016 Jan 13;11(1):e0146009. doi: 10.1371/journal.pone.0146009 (PMC4712000; doi:10.1371/journal.pone.0146009)

Recruitment (standardized)

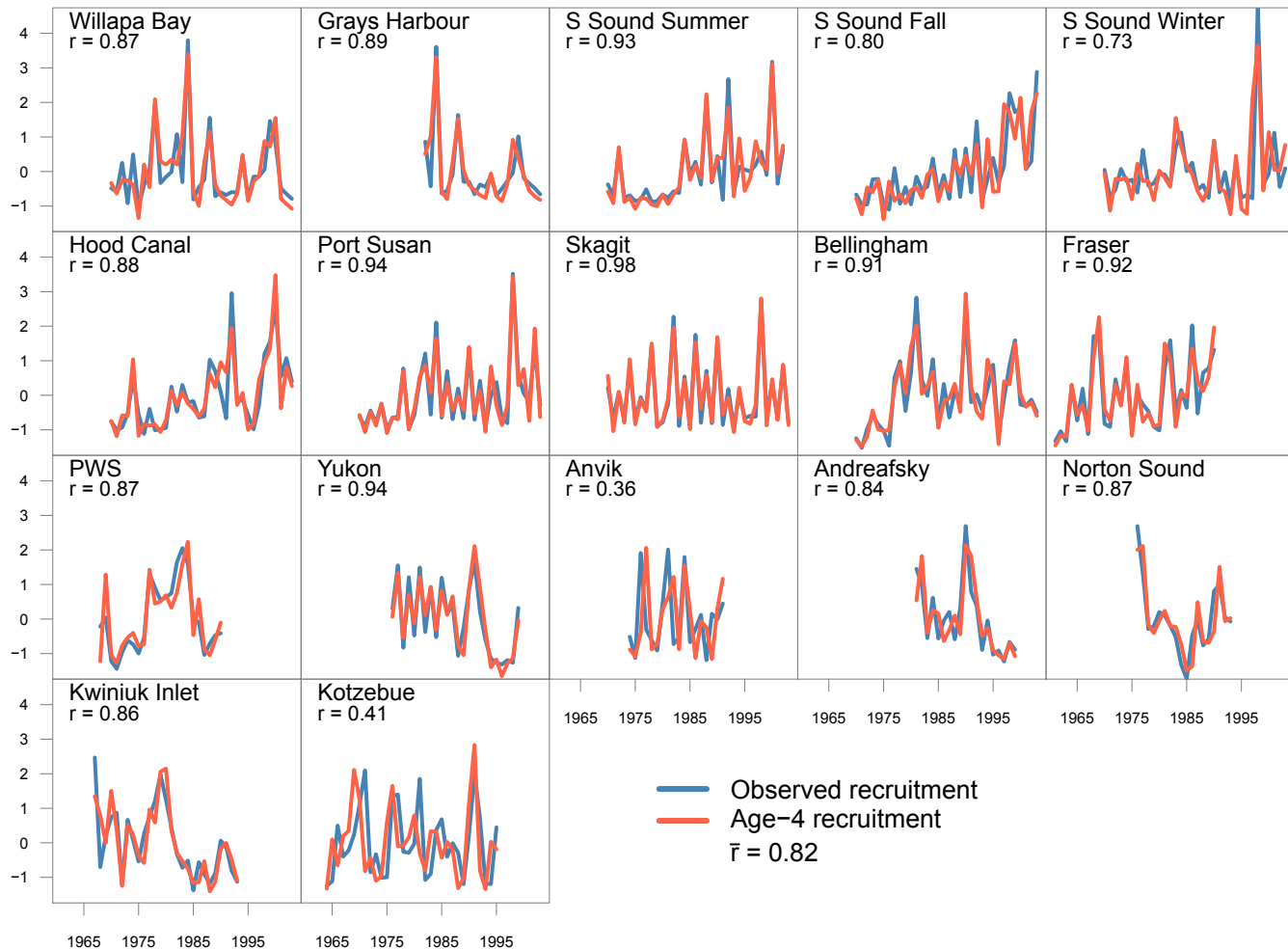

Brood year

Supplement: S1 Fig — Recruitment series are standardized to a mean of 0 and standard deviation of 1. Correlation coefficients between the two recruitment series are given within each panel (r) and r¯ gives the average correlation across the 17 stocks. (PDF) [file pone.0146009.s001.pdf]

-2 -1 0 1 2 Standard deviations

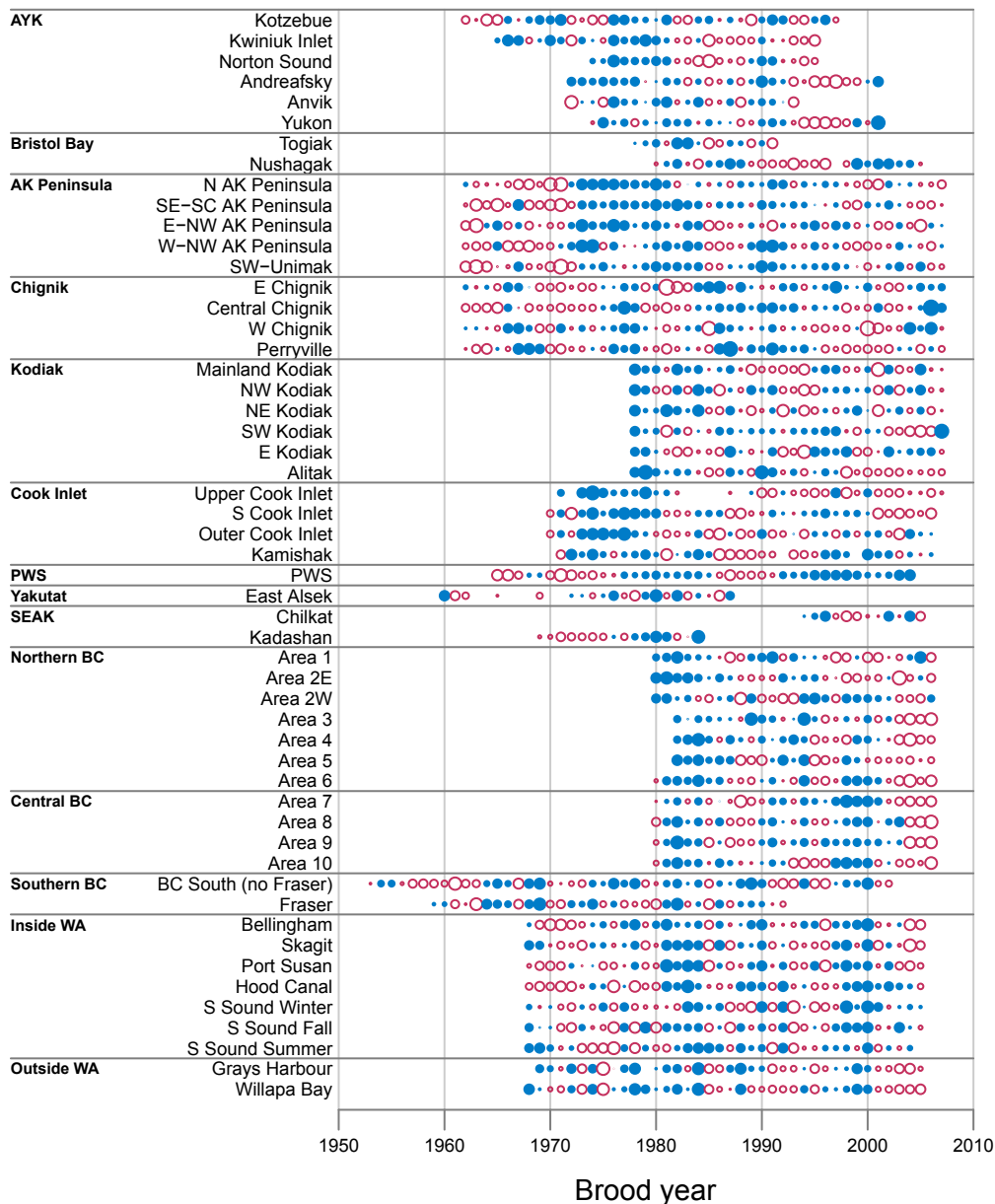

Supplement: S2 Fig — The residual series are standardized to a mean of 0 and standard deviation of 1. The ordinate gives the stock, which are arranged south (bottom) to north (top) and grouped by geographic region. The area of the circle indicates the magnitude of the productivity values. Open circles represent negative values (red) and filled circles indicate positive values (blue). (PDF) [file pone.0146009.s002.pdf]

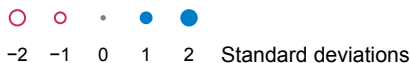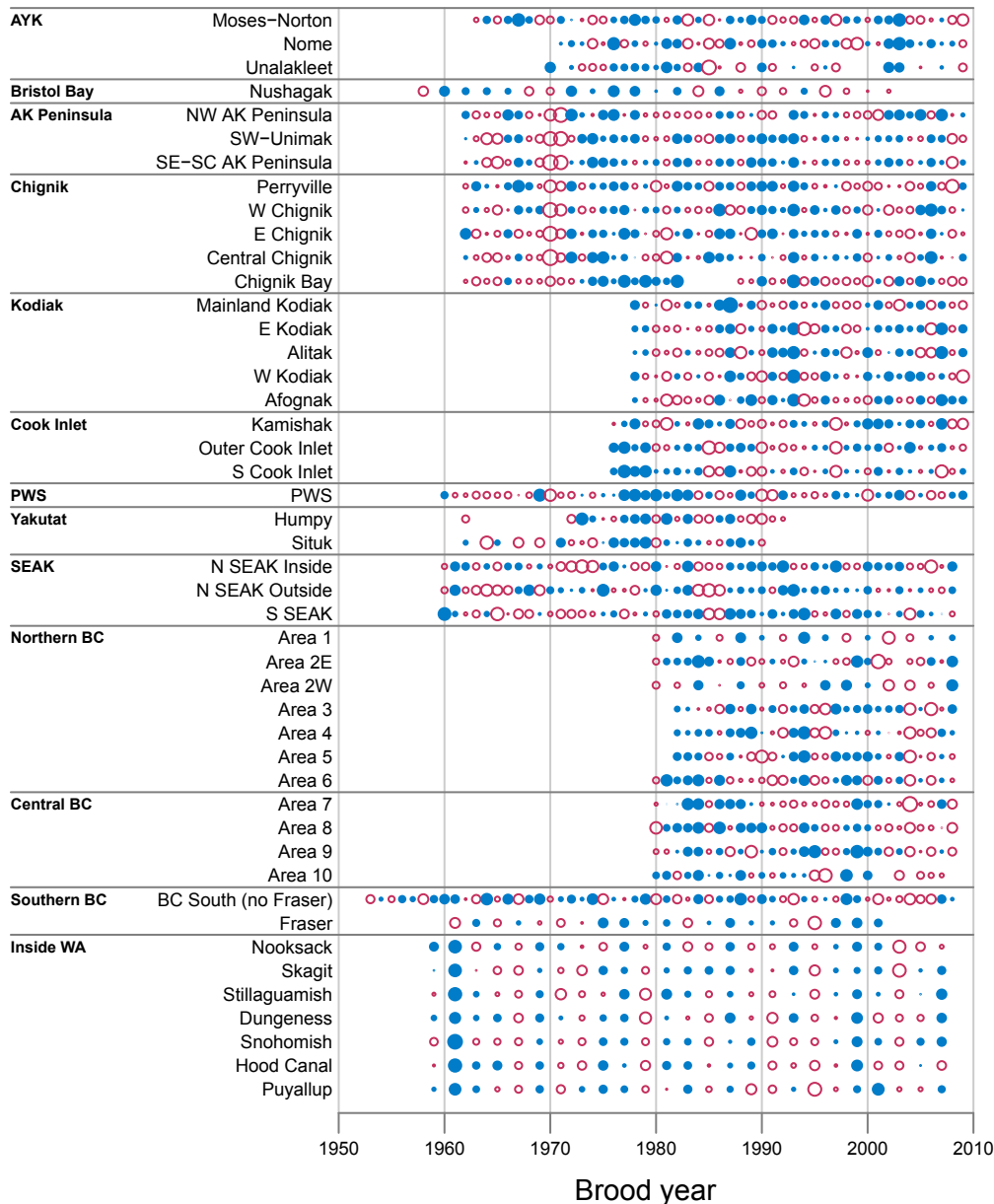

Supplement: S3 Fig — The residual series are standardized to a mean of 0 and standard deviation of 1. The ordinate gives the stock, which are arranged south (bottom) to north (top) and grouped by geographic region. The area of the circle indicates the magnitude of the productivity values. Open circles represent negative values (red) and filled circles indicate positive values (blue). (PDF) [file pone.0146009.s003.pdf]

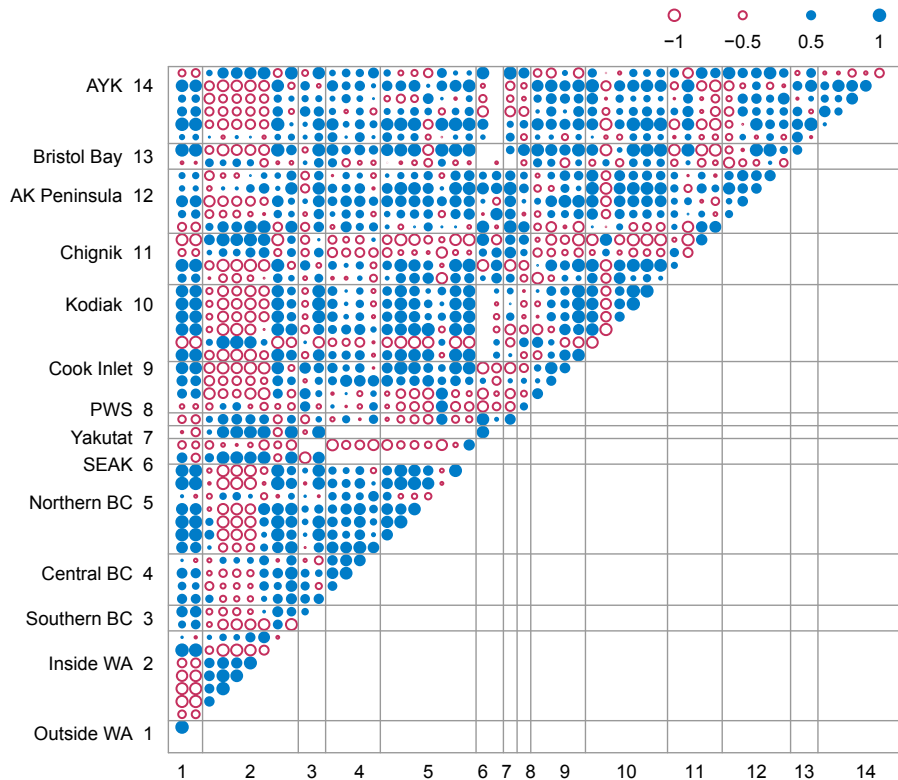

Supplement: S4 Fig — Correlations were calculated using all available brood years. The magnitude of the correlation is given by the area of the circle with larger circles representing larger correlations than smaller circles. Negative correlations between stocks are shown as open circles (red) and positive correlations are shown as filled circles (blue). Stocks are grouped by geographic region, which are arranged south (bottom, left) to north (top, right). (PDF) [file pone.0146009.s004.pdf]

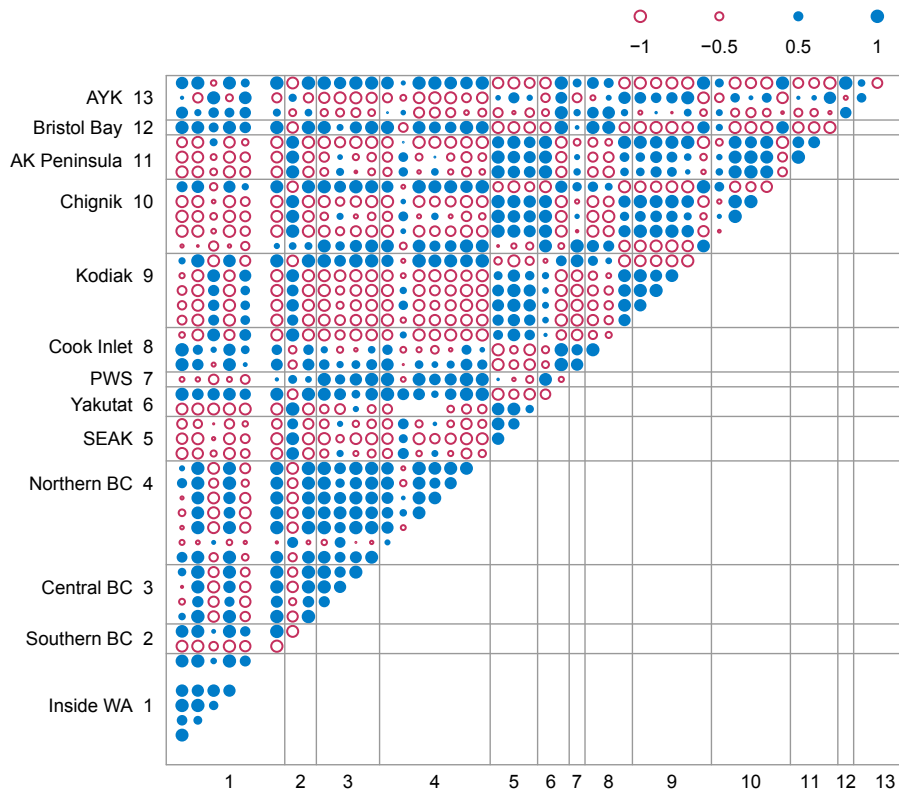

Supplement: S5 Fig — Correlations were calculated using all available brood years. The magnitude of the correlation is given by the area of the circle with larger circles representing larger correlations than smaller circles. Negative correlations between stocks are shown as open circles (red) and positive correlations are shown as filled circles (blue). Stocks are grouped by geographic region, which are arranged south (bottom, left) to north (top, right). (PDF) [file pone.0146009.s005.pdf]

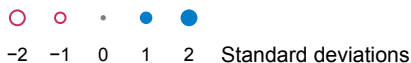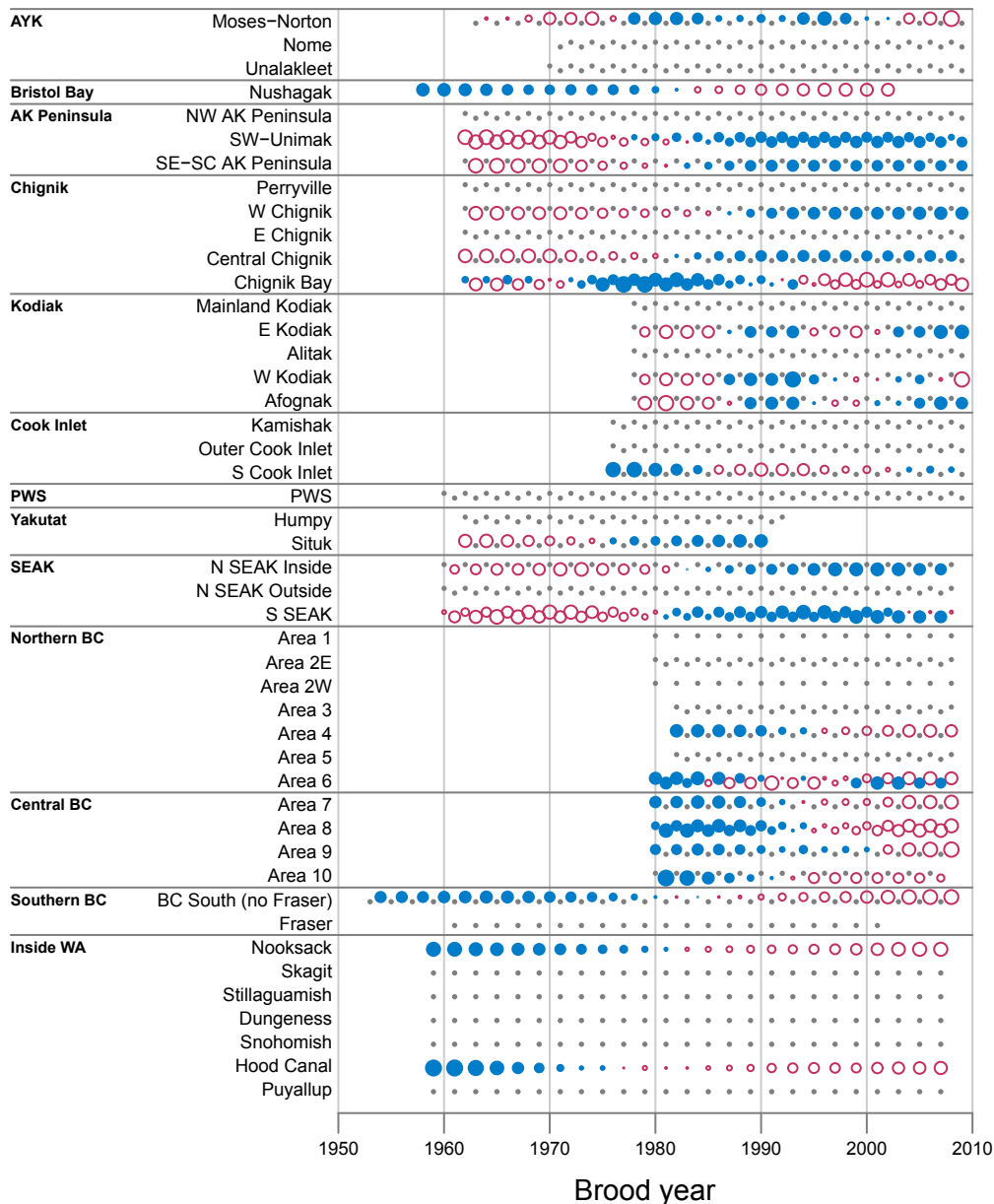

Supplement: S6 Fig — Each time series shows the smoothed αt estimates from the Kalman filter model where each series is standardized to a mean of 0 and standard deviation of 1. Even and odd year brood lines are shown concurrently for each stock with the even year brood lines offset slightly above the odd year brood lines. The ordinate gives the stock, which are arranged south (bottom) to north (top) and grouped by geographic region. The area of the circle indicates the magnitude of the productivity values. Open circles represent negative values (red) and filled circles indicate positive values (blue). Small solid grey dots indicate zero values, whereas missing values are not shown. (PDF) [file pone.0146009.s006.pdf]

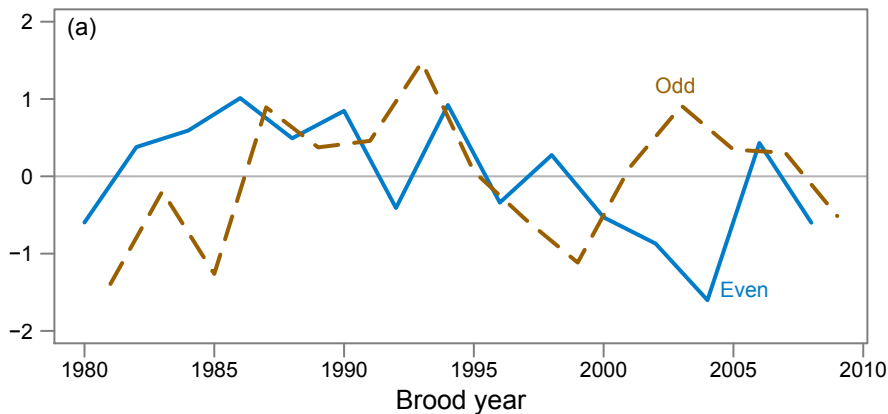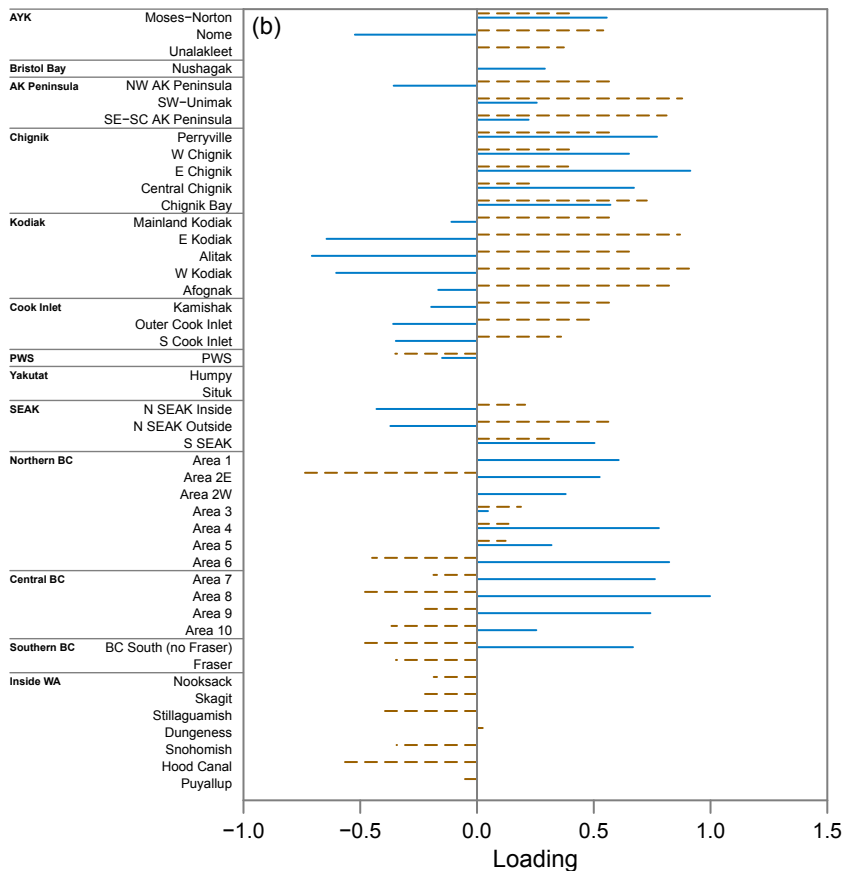

Supplement: S7 Fig — (a) Even brood year (solid line) and odd brood year (dashed line) common productivity trends and (b) stock specific loadings for the even year (solid horizontal bars) and odd year (dashed horizontal bars) common trends. In the bottom panel, stocks are ordered south (bottom) to north (top) and are grouped by geographic region. (PDF) [file pone.0146009.s007.pdf]

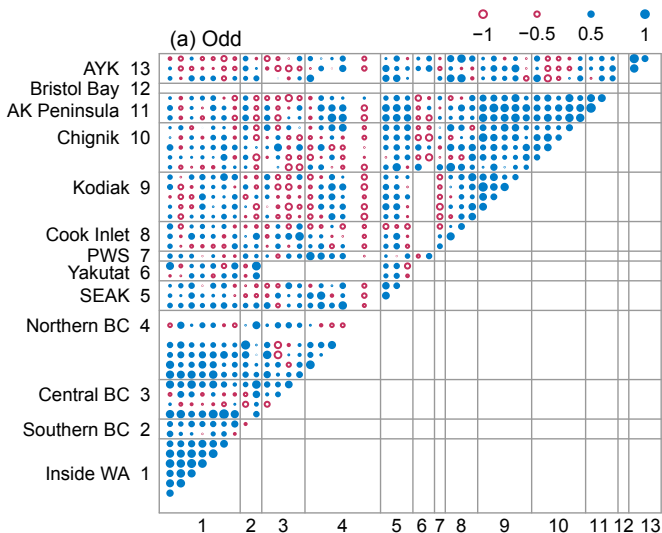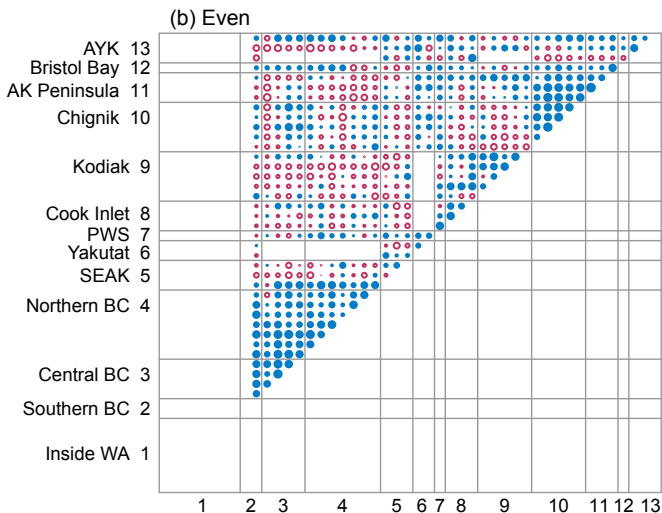

Supplement: S8 Fig — (a) between stock correlation coefficients for odd year runs and (b) correlations for even year runs. The magnitude of the correlation is given by the area of the circle with larger circles representing larger correlations than smaller circles. Negative correlations between stocks are shown as open circles (red) and positive correlations are shown as filled circles (blue). Stocks are grouped by geographic region, which are arranged south (bottom, left) to north (top, right). (PDF) [file pone.0146009.s008.pdf]
