# Supplementary material for: Regional-Scale Declines in Productivity of Pink and Chum Salmon Stocks in Western North America
Source: PLoS One. 2016 Jan 13;11(1):e0146009. doi: 10.1371/journal.pone.0146009 (PMC4712000; doi:10.1371/journal.pone.0146009)
Supplement: S1 Table — Brood years gives the range of years available for each stock; N gives the total brood years with data; R/S gives the average spawner to recruit ratio over all available brood years; Stationary α and β give the parameter estimates from the best fit stationary Ricker model; Kalman filter αt gives the average αt value and SD gives the standard deviation for the αt series; Kalman filter S/N gives the signal-to-noise ratio for that stock. (PDF) [file pone.0146009.s009.pdf]

Table S1. Chum salmon data set summary. Brood years gives the range of years available for each stock; N gives the total brood years with data; R/S gives the average spawner to recruit ratio over all available brood years; Stationary α and β give the parameter estimates from the best fit stationary Ricker model; Kalman filter α_t gives the average α_t value and SD gives the standard deviation for the α_t series; Kalman filter S/N gives the signal-to-noise ratio for that stock.

					Stationary		Kalman filter			
Region	Stock	Brood years	N	R/S	α	β	α_t	SD	S/N	Source[a]
Outside WA	Willapa Bay	1968-2005	38	2.2	1.18	-25.17	1.11	0.10	0.007	1
	Grays Harbour	1969-2005	36	2.3	1.02	-31.46	0.98	0.07	0.004	1
Inside WA	South Sound Summer	1968-2004	37	3.6	1.36	-22.31	1.40	0.30	0.112	1
	South Sound Fall	1968-2005	38	3.9	1.58	-3.90	1.70	0.14	0.012	1
	South Sound Winter	1968-2005	38	2.5	1.08	-13.96	1.07	0.00	0.000	1
	Hood Canal	1968-2005	38	5.7	2.00	-12.45	2.21	0.45	0.078	1
	Port Susan[b]	1968-2005	38	2.5	0.98	-4.39	0.99	0.00	0.000	1
	Skagit	1968-2005	38	2.2	0.83	-4.79	0.84	0.00	0.000	1
	Bellingham[c]	1968-2005	38	2.8	1.46	-21.86	1.49	0.28	0.184	1
Southern BC	Fraser	1959-1992	34	2.3	1.00	-0.66	0.99	0.00	0.000	2
	BC South (no Fraser River)[d]	1953-2002	50	2.0	1.08	-0.79	1.16	0.11	0.014	3, 4
Central BC	Statistical Area 10	1980-2006	27	1.9	0.87	-19.46	0.99	0.83	1.959	5, 6
	Statistical Area 9	1980-2006	27	2.0	0.94	-11.12	0.56	1.01	1.554	5, 6
	Statistical Area 8	1980-2006	27	2.9	1.85	-3.49	1.38	0.60	0.882	5, 6
	Statistical Area 7	1980-2006	27	1.8	1.86	-5.20	1.94	0.44	3.561	5, 6
Northern BC	Statistical Area 6	1980-2006	27	2.0	0.95	-3.12	0.95	0.70	0.783	5, 6
	Statistical Area 5	1982-2006	25	2.2	1.49	-101.91	1.62	0.23	0.048	5, 6
	Statistical Area 4	1982-2006	25	2.1	0.68	-15.93	0.74	0.63	0.213	5, 6
	Statistical Area 3	1982-2006	25	2.3	1.55	-13.84	1.52	0.40	0.229	5, 6
	Statistical Area 2W	1980-2006	27	1.5	1.37	-10.59	1.38	0.00	0.000	5, 6
	Statistical Area 2E	1980-2006	27	1.4	0.09	0.12	1.03	0.57	0.253	5, 6
	Statistical Area 1	1980-2006	27	3.7	1.22	-25.20	1.52	0.54	0.078	5, 6
Southeast Alaska	Kadashan	1969-1984	16	3.0	1.90	-49.48	1.73	0.64	0.576	7
	Chilkat	1994-2005	12	2.8	1.70	-5.05	1.70	0.00	0.000	7
Yakutat	East Alsek	1960-1987	21	3.8	1.76	-72.26	1.71	0.00	0.000	7
Prince William Sound	Prince William Sound	1965-2004	40	8.9	2.67	-3.76	2.61	0.69	>20	8

Continued on next page

Region	Stock	Brood years	N	R/S	Stationary		Kalman filter			Source[a]
					α	β	α_t	SD	S/N	
Cook Inlet	Kamishak	1971-2006	34	2.1	1.23	-8.21	1.39	0.30	0.162	9, 10
	Outer Cook Inlet	1970-2006	37	2.2	0.82	-12.55	0.93	0.36	0.093	9, 10
	Southern Cook Inlet	1970-2006	37	4.5	2.03	-312.83	2.07	0.40	0.432	9, 10
	Upper Cook Inlet	1971-2007	31	3.3	1.44	-83.92	1.39	0.46	0.087	11
Kodiak	Alitak	1978-2007	30	3.9	1.79	-18.68	1.92	0.32	0.140	12
	East Kodiak	1978-2007	30	4.4	1.97	-9.26	1.95	0.00	0.000	12
	Southwest Kodiak	1978-2007	30	5.5	1.76	-21.20	1.94	0.16	0.017	12
	Northeast Kodiak	1978-2007	30	3.3	1.58	-52.11	1.68	0.23	0.043	12
	Northwest Kodiak	1978-2007	30	5.3	2.16	-7.22	2.15	0.00	0.000	12
	Mainland Kodiak	1978-2007	30	2.3	1.46	-4.43	1.55	0.17	0.044	12
Chignik	Perryville	1962-2007	46	3.8	1.15	-9.54	1.12	0.42	0.240	13, 14
	Western Chignik	1962-2007	46	5.5	1.91	-23.26	1.91	0.00	0.000	13, 14
	Central Chignik	1962-2007	46	4.2	1.13	-12.54	1.33	0.61	0.456	13, 14
	Eastern Chignik	1962-2007	46	2.4	1.30	-8.48	1.34	0.12	0.006	13, 14
Alaska Peninsula	Southwest Unimak[e]	1962-2007	46	2.6	1.29	-3.09	1.84	0.55	0.271	12
	Western-Northwest District[f]	1962-2007	46	2.6	1.03	-9.78	0.99	0.49	1.196	12
	Eastern-Northwest District[g]	1962-2007	46	1.7	0.88	-3.26	0.92	0.20	0.074	12
	SE and SC Districts[h]	1962-2007	46	2.7	1.39	-1.77	1.55	0.29	0.244	12
	Northern AK District	1962-2007	46	2.4	1.17	-3.55	1.19	0.54	2.172	12
Bristol Bay	Nushagak	1980-2005	25	2.7	1.18	-0.94	1.21	0.33	1.090	15
	Togiak	1978-1991	14	2.7	2.07	-5.93	2.17	0.18	0.103	15
AYK[i]	Yukon[j]	1974-2001	28	1.9	0.94	-0.91	0.68	0.59	15.073	16, 17
	Anvik	1972-1993	22	1.9	1.13	-1.02	1.22	0.00	0.000	16, 18
	Andreafsky	1972-2001	30	1.5	0.81	-8.02	0.74	0.60	1.379	16, 19
	Norton Sound[k]	1974-1995	22	1.8	1.43	-25.89	1.43	0.42	4.934	16, 20
	Kwiniuk Inlet[l]	1965-1995	31	2.1	1.18	-17.59	1.19	0.43	0.203	16, 21
	Kotzebue	1962-1997	36	2.0	1.17	-1.84	1.15	0.00	0.000	16, 22

[a] 1: Jeff Haymes, Washington Department of Fish and Wildlife, Olympia, WA; 2: Ryall et al. (1999); 3: Pieter Van Will, Fisheries and Oceans Canada (DFO), Port Hardy, BC; 4: Van Will et al. (2009); 5: David Peacock, DFO, Prince Rupert, BC; 6: English et al. (2011); 7: Steve Heinl, Alaska Department of Fish and Game (ADFG), Ketchikan, AK; 8: Botz et al. (2012); 9: Ted Otis, ADFG, Homer, AK; 10: Hollowell et al. (2012); 11: Patrick Shields, ADFG, Soldotna, AK; 12: Matt Foster, ADFG,

Kodiak, AK; 13: Charles Russell, ADFG, Kodiak, AK; 14: Owen and Sarafin (1999); 15: Lowell Fair, ADFG, Anchorage, AK; 16: Doug Eggers, ADFG, Juneau, AK; 17: Fleischman and Borba (2009); 18: Clark and Sandone (2001); 19: Fleischman and Evenson (2010); 20: Clark (2001a); 21: Clark (2001b); 22: Eggers and Clark (2006).

[b] Sum of Stillaguamish and Snohomish data sets

[c] Sum of Nooksack and Samish data sets

[d] Statistical Areas 11-17, 28A, 28B, 29B

[e] Sum of Southwestern and Unimak Districts

[f] Sum of Dublin, Bechevin & Urilia Bays, and Swanson Lagoon data sets

[g] Sum of Izembek and Moffet Bay data sets

[h] Sum of Southeastern and Southcentral Districts

[i] Arctic Yukon Kuskokwim

[j] Fall run only

[k] District 1

[l] Sum of Kwiniuk and Tubutulik Rivers data sets

References

Botz, J., G. Hollowell, T. Sheridan, R. Brenner, and S. Moffitt. 2012. 2010 Prince William Sound area finfish management report. Technical report, Fishery Management Report No. 12-06, Alaska Department of Fish and Game.

Clark, J. 2001a. Biological escapement goal for chum salmon in subdistrict one of Norton Sound. Technical report, Regional Information Report No. 3AO1-09, Alaska Department of Fish and Game.

Clark, J. 2001b. Biological escapement goals for Kwiniuk and Tubutulik chum salmon. Technical report, Regional Information Report No. 3AOI-08, Alaska Department of Fish and Game.

Clark, J. and G. Sandone. 2001. Biological escapement goal for Anvik River chum salmon. Technical report, Regional Information Report No. 3AO1-06, Alaska Department of Fish and Game.

Eggers, D. and J. Clark. 2006. Assessment of historical runs and escapement goals for Kotzebue area chum salmon. Technical report, Fishery Manuscript No. 06-01, Alaska Department of Fish and Game.

English, K., T. Mochizuki, and D. Robichaud. 2011. Review of North and Central Coast salmon indicator streams and estimating escapement, catch and run size for each salmon conservation unit. Technical report, LGL Limited.

Fleischman, S. and B. Borba. 2009. Escapement estimation, spawner-recruit analysis, and escapement goal recommendation for fall chum salmon in the Yukon River drainage. Technical report, Fishery Manuscript Series No. 09-08, Alaska Department of Fish and Game.

Fleischman, S. and D. Evenson. 2010. Run reconstruction, spawner-recruit analysis, and escapement goal recommendation for summer chum salmon in the east fork of the Andreafsky River. Technical report, Fishery Manuscript Series No. 10-04, Alaska Department of Fish and Game.

Hollowell, G., T. Otis, and E. Ford. 2012. 2011 Lower Cook Inlet area finfish management report. Technical report, Fishery Management Report No. 12-30, Alaska Department of Fish and Game.

Owen, D. and D. Sarafin. 1999. Chignik management area annual finfish management report, 1996. Technical report, Regional Information Report No. 4K99-33, Alaska Department of Fish and Game.

Ryall, P., C. Murray, V. Palermo, D. Bailey, and D. Chen. 1999. Status of Clockwork chum salmon stock and review of the Clockwork management strategy. Technical report, Canadian Department of Fisheries and Oceans, Canadian Stock Assessment Secretariat Research Document 99/169, Ottawa.

Van Will, P., R. Brahniuk, L. Hop Wo, and G. Pestal. 2009. Certification unit profile: Inner South Coast chum salmon (excluding Fraser River). Technical report, Canadian Manuscript Report of Fisheries and Aquatic Sciences 2876.
