# Supplementary material for: Regional-Scale Declines in Productivity of Pink and Chum Salmon Stocks in Western North America
Source: PLoS One. 2016 Jan 13;11(1):e0146009. doi: 10.1371/journal.pone.0146009 (PMC4712000; doi:10.1371/journal.pone.0146009)
Supplement: S2 Table — Brood years gives the range of years available for each stock; N gives the total brood years with data; R/S gives the average spawner to recruit ratio over all available brood years; Stationary α and β give the parameter estimates from the best fit stationary Ricker model; Kalman filter αt gives the average αt value and SD gives the standard deviation for the αt series; Kalman filter S/N gives the signal-to-ratio for that stock. (PDF) [file pone.0146009.s010.pdf]

Table S2. Pink salmon data set summary. Brood years gives the range of years available for each stock; N gives the total brood years with data; R/S gives the average spawner to recruit ratio over all available brood years; Stationary α and β give the parameter estimates from the best fit stationary Ricker model; Kalman filter α_t gives the average α_t value and SD gives the standard deviation for the α_t series; Kalman filter S/N gives the signal-to-ratio for that stock.

					Stationary		Kalman filter			
Region	Stock	Brood years	N	R/S	α	β	α_t	SD	S/N	Source[a]
Inside WA	Puyallup[b]	1959-2007	25	3.0	0.77	-0.42	0.77	0.00	0.000	1
	Hood Canal[b, c]	1959-2007	25	2.9	0.59	-4.35	0.84	0.54	0.047	1
	Snohomish[b]	1959-2007	25	2.6	0.81	-0.76	0.84	0.00	0.000	1
	Dungeness[b]	1959-2007	25	2.7	0.53	-5.84	0.54	0.00	0.000	1
	Stillaguamish[b]	1959-2007	25	3.0	1.13	-3.32	1.16	0.00	0.000	1
	Skagit[b]	1959-2007	25	3.2	1.72	-2.64	1.75	0.00	0.000	1
	Nooksack[b]	1959-2007	25	3.0	1.53	-14.52	1.50	0.02	0.001	1
Southern BC	Fraser[b]	1961-2001	21	4.2	1.84	-0.15	1.87	0.00	0.000	2
	BC South (no Fraser River)[d]	1953-2008	56	2.4	1.16	-0.52	1.18	0.00	0.000	3
Central BC	Statistical Area 10	1980-2007	23	4.0	1.16	-22.85	1.10	0.00	0.000	4
	Statistical Area 9	1980-2008	29	1.9	0.35	-0.75	0.37	0.00	0.000	4
	Statistical Area 8	1980-2008	29	1.8	0.86	-0.28	1.04	0.33	0.042	4
	Statistical Area 7	1980-2008	29	1.6	0.92	-1.77	0.91	0.18	0.033	4
Northern BC	Statistical Area 6	1980-2008	29	2.3	0.63	-0.09	0.61	0.23	0.042	4
	Statistical Area 5	1982-2008	27	1.9	0.98	-1.93	0.98	0.00	0.000	4
	Statistical Area 4	1982-2008	27	3.7	1.40	-0.27	1.55	0.27	0.023	4
	Statistical Area 3	1982-2008	27	2.2	1.11	-0.64	1.10	0.00	0.000	4
	Statistical Area 2W[e]	1980-2008	15	2.1	1.12	-3.06	1.14	0.00	0.000	4
	Statistical Area 2E	1980-2008	28	1.3	-0.07	0.10	-0.07	0.06	0.006	4
	Statistical Area 1[e]	1980-2008	15	1.8	0.96	-0.58	1.09	0.00	0.000	4
Southeast Alaska	Southern SEAK[f]	1960-2008	49	2.8	1.22	-0.03	1.64	0.33	0.061	5, 6
	Northern SEAK Outside[g]	1960-2008	49	1.9	0.54	-0.03	0.77	0.25	0.038	5, 6
	Northern SEAK Inside[h]	1960-2008	49	2.3	0.76	-0.02	1.18	0.35	0.105	5, 6
Yakutat	Situk	1962-1990	25	2.2	1.41	-7.32	1.49	0.34	0.038	5, 7
	Humpy	1962-1992	22	3.9	1.39	-17.56	1.43	0.00	0.000	5, 7
Prince William Sound	Prince William Sound	1960-2009	50	2.6	1.11	-0.08	1.11	0.00	0.000	8

Continued on next page

Region	Stock	Brood years	N	R/S	Stationary		Kalman filter			Source[a]
					α	β	α_t	SD	S/N	
Cook Inlet	Southern Cook Inlet[i]	1976-2009	34	1.7	0.71	-5.37	0.98	0.40	0.097	9
	Outer Cook Inlet[j]	1976-2009	34	2.9	1.10	-1.70	1.08	0.00	0.000	9
	Kamishak District[k]	1976-2009	34	3.3	1.00	-2.77	0.99	0.00	0.000	9
Kodiak	Afognak District	1978-2009	32	3.4	2.27	-1.89	2.24	0.11	0.018	10
	Westside Kodiak	1978-2009	32	3.0	1.40	-0.12	1.41	0.00	0.000	10
	Alitak District	1978-2009	32	2.8	1.22	-0.35	1.22	0.00	0.000	10
	Eastside Kodiak	1978-2009	32	2.2	0.83	-0.11	0.83	0.00	0.000	10
	Mainland Kodiak	1978-2009	32	1.7	0.68	-0.24	0.68	0.01	0.001	10
Chignik	Chignik Bay	1962-2009	43	18.4	2.47	-22.56	2.31	0.71	0.147	11, 12
	Central Chignik	1962-2009	48	2.9	0.83	-1.13	0.93	0.17	0.007	11, 12
	Eastern Chignik	1962-2009	48	1.8	0.69	-0.83	0.70	0.00	0.000	11, 12
	Western Chignik	1962-2009	48	5.4	1.96	-5.66	1.94	0.00	0.000	11, 12
	Perryville	1962-2009	48	2.6	0.97	-2.67	0.95	0.14	0.007	11, 12
Alaska Peninsula	SE and SC Districts[l]	1962-2009	48	3.6	1.34	-0.23	1.70	0.44	0.063	10
	Southwest Unimak[m]	1962-2009	48	3.5	1.17	-0.51	1.74	0.66	0.128	10
	Northwestern District	1962-2009	47	4.3	0.81	-8.46	0.80	0.00	0.000	10
Bristol Bay	Nushagak[e]	1958-2002	23	3.9	0.96	-0.35	1.03	0.31	0.015	13
AYK[n]	Unalakleet	1970-2009	28	13.0	1.72	-8.96	1.57	0.00	0.000	14, 15
	Nome	1971-2009	39	7.4	0.86	-1.90	0.86	0.00	0.000	14, 15
	Moses-Norton[o]	1963-2009	47	2.9	0.65	-1.53	0.66	0.00	0.000	14, 15

[a] 1: Jeff Haymes, Washington Department of Fish and Wildlife, Olympia, WA; 2: Pacific Salmon Commission (2012); 3: Pieter Van Will, Fisheries and Oceans Canada (DFO), Port Hardy, BC; 4: David Peacock, DFO, Prince Rupert, BC; 5: Steve Heinl, Alaska Department of Fish and Game (ADFG), Ketchikan, AK; 6: Piston and Heinl (2011); 7: Clark (1995); 8: Steve Moffitt, ADFG, Cordova, AK; 9: Ted Otis, ADFG, Homer, AK; 10: Matt Foster, ADFG, Kodiak, AK; 11: Charles Russell, ADFG, Kodiak, AK; 12: Owen and Sarafin (1999); 13: Lowell Fair, ADFG, Anchorage, AK; 14: Jenefer Bell, ADFG, Nome, AK; 15: Menard et al. (2011).

[b] Data were only available for odd year brood lines because few if any pink salmon spawn in even years.

[c] Sum of Dosewallips, Duckabush, and Hamma Hamma data sets

[d] Statistical Areas 11-16; Excludes Fraser River

[e] Data were only available for even years because few if any pink salmon spawn in odd years

[f] Districts 101-108

[g] Districts 109-112, 114, 115
[h] District 113
[i] Sum of Humpy Creek, Seldovia Bay data sets
[j] Sum of Port Chatham, Port Dick, Rocky River, Windy Creek, and South Nuka data sets
[k] Sum of Bruin River, Sunday Creek, and Brown's Peak Creek data sets
[l] Sum of Southeastern and Southcentral Districts
[m] Sum of Southwestern and Unimak Districts
[n] Arctic Yukon Kuskokwim
[o] Sum of Moses Point and Norton Bay Subdistricts

References

Clark, J. H. 1995. Biological escapement goals for even and odd-year pink salmon returning to the Situk River and to Humpy Creek near Yakutat Alaska. Alaska Department of Fish and Game, Regional Information Report No. 1J95-08 .

Menard, J., J. Soong, and S. Kent. 2011. 2009 annual management report Norton Sound, Port Clarence, and Kotzebue. Technical report, Fishery Management Report No. 11-46, Alaska Department of Fish and Game.

Owen, D. and D. Sarafin. 1999. Chignik management area annual finfish management report, 1996. Technical report, Regional Information Report No. 4K99-33, Alaska Department of Fish and Game.

Pacific Salmon Commission. 2012. Report of the Fraser River Panel to the Pacific Salmon Commission on the 2007 Fraser River sockeye and pink salmon fishing season. Technical report, Pacific Salmon Commission.

Piston, A. W. and S. C. Heinl. 2011. Pink salmon stock status and escapement goals in Southeast Alaska. Alaska Department of Fish and Game, Special Publication No. 11-18 .
